# Trends in Gender and Racial/Ethnic Disparities in Physical Disability and Social Support Among U.S. Older Adults With Cognitive Impairment Living Alone, 2000–2018

**DOI:** 10.1093/geroni/igad028

**Published:** 2023-03-21

**Authors:** Shanquan Chen, Huanyu Zhang, Benjamin R Underwood, Dan Wang, Xi Chen, Rudolf N Cardinal

**Affiliations:** Department of Psychiatry, University of Cambridge, Cambridge, UK; International Centre for Evidence in Disability, London School of Hygiene & Tropical Medicine, London WC1E 7HT, UK; Shenzhen Research Institute, The Chinese University of Hong Kong, Shenzhen, China; Department of Psychiatry, University of Cambridge, Cambridge, UK; Cambridgeshire & Peterborough NHS Foundation Trust, Fulbourn, Cambridge, UK; Faculty of Health Sciences, Ontario Tech University, Oshawa, Ontario, Canada; Centre for Disability Prevention and Rehabilitation, Ontario Tech University, Oshawa, Ontario, Canada; School of Public Health, Yale University, New Haven, Connecticut, USA; Department of Psychiatry, University of Cambridge, Cambridge, UK; Cambridgeshire & Peterborough NHS Foundation Trust, Fulbourn, Cambridge, UK

**Keywords:** Cognitive impairment, Gender disparity, Physical disability, Racial/ethnic disparity, Social support

## Abstract

**Background and Objectives:**

Informal care is the primary source of support for older adults with cognitive impairment, yet is less available to those who live alone. We examined trends in the prevalence of physical disability and social support among older adults with cognitive impairment living alone in the United States.

**Research Design and Methods:**

We analyzed 10 waves of data from the U.S. Health and Retirement Survey spanning 2000–2018. Eligible people were those aged ≥65, having cognitive impairment, and living alone. Physical disability and social support were measured via basic and instrumental activities of daily living (BADLs, IADLs). We estimated linear temporal trends for binary/integer outcomes via logistic/Poisson regression, respectively.

**Results:**

A total of 20 070 participants were included. Among those reporting BADL/IADL disability, the proportion unsupported for BADLs decreased significantly over time (odds ratio [OR] 0.98, 95% confidence interval [CI] 0.97–0.99), and the proportion unsupported for IADLs increased (OR = 1.02, CI 1.01–1.04). Among those receiving IADL support, the number of unmet IADL support needs increased significantly over time (relative risk [RR] 1.04, CI 1.03–1.05). No gender disparities were found for these trends. Over time, Black respondents had a relatively increasing trend of being BADL-unsupported (OR = 1.03, CI 1.0–1.05) and Hispanic and Black respondents had a relatively increasing trend in the number of unmet BADL needs (RR = 1.02, CI 1.00–1.03; RR = 1.01, CI 1.00–1.02, respectively), compared to the corresponding trends in White respondents.

**Discussion and Implications:**

Among lone-dwelling U.S. older adults with cognitive impairment, fewer people received IADL support over time, and the extent of unmet IADL support needs increased. Racial/ethnic disparities were seen both in the prevalence of reported BADL/IADL disability and unmet BADL/IADL support needs; some but not all were compatible with a reduction in disparity over time. This evidence could prompt interventions to reduce disparities and unmet support needs.

**Translational Significance:** This study demonstrates that among lone-dwelling cognitively impaired U.S. older adults, although the overall prevalence of basic and instrumental activities of daily living (BADL and IADL) disability remained steady from 2000 to 2018, fewer people received IADL support and the extent of unmet IADL support needs increased over time. Both gender and racial/ethnic differences were seen in BADL/IADL needs and support. Some racial/ethnic disparities narrowed, for example, with unmet BADL support needs worsening over time in minority ethnicity groups (Hispanic and Black) relative to the majority (White) ethnic group but from a better baseline. Data such as these allow for identifying groups most in need and customized interventions.

Cognitive impairment, including memory loss and other cognitive dysfunction, may form part of a dementia syndrome or prodrome. The majority of cases of dementia are caused by Alzheimer’s disease (1). It was estimated that 58 million people in the United States had Alzheimer’s disease in 2021, and this number is projected to reach 88 million by 2050 (1). The American Academy of Neurology estimated that about 8% of people aged 65–69 have a mild cognitive impairment, about 15% of those aged 75–79, about 25% of those aged 80–84, and about 37% of people 85 years of age and older (2).

Cognitive impairment is associated with functional impairment in daily life, independent of the effects of depression, fatigue, and motor disability (3). Deficits in cognitive ability can impair day-to-day decision making, motivation, and new learning sufficient to affect self-care in both higher-order and basic activities of daily living as well as to affect capacity for gainful employment and promote the transition to permanent disability status (3–5). A recent U.S. study indicated that nearly 70% of people with cognitive impairment developed physical disability over 10 years of follow-up, which may be a further cause of impairment in daily living (6). With an increasingly aging society, cognitive impairment and its associated care needs are likely to become a greater public health problem.

Currently, informal care (mainly from families and friends) is the primary source of care for cognitively impaired Americans, accounting for 83% of all care (1). However, this form of care is often not available for those who live alone, as people living alone experience greater isolation associated with a diminished social network of available family or friend caregivers (1). Older adults living alone have significantly more unmet needs in the domains of housework and community living and are at greater risks of adverse health outcomes compared with those living with others (7–9). Given that a considerable proportion of the older adults lives alone (almost one third of U.S. older adults with cognitive impairment) (5), meeting the needs of cognitively impaired U.S. older adults living alone is an important issue.

Gender and racial disparities in the prevalence of cognitive impairment and corresponding physical disabilities and social support were widely documented (5,10–17). For instance, Mexican American older adults who live alone experience dual risks of both greater cognitive impairment and receiving low support from others when compared to Mexican American older adults who live with others (15); compared to White Americans, Black and Hispanic Americans were reported to have a higher prevalence of dementia and less access to health services (10,11,16); females were more likely to experience racial/ethnic differences in physical disabilities and corresponding support among older adults living alone with cognitive impairment than males (17). Recent studies (12,18) also estimated the time trend of gender and racial/ethnic disparities on the prevalence of cognitive impairment; however, to the best of our knowledge, the time trend in physical disabilities and social support has not been quantified over time.

This study aimed to examine temporal trends in the prevalence of physical disability and social support among older adults living alone with cognitive impairment from 2000 to 2018 in the United States, with a focus on gender and racial/ethnic disparities. Such evidence might be expected to help address the concerns of cognitively impaired older adults living alone via targeting vulnerable subgroups and supporting the development of interventions and public policies to eliminate inequalities (8,10). We hypothesized that (i) the prevalence of physical disability would increase over time; (ii) the probability of receiving no social support would decrease over time; and (iii) gender and racial/ethnic disparities may exist in the above trends.

## Method

### Data Source and Participants

This study used data from the Health and Retirement Survey (HRS), a nationally representative and biennial study of U.S. adults aged 50 years or older. Each participant completed a standardized questionnaire, face-to-face or via internet/telephone assessments, described elsewhere (19). Data included sociodemographic characteristics, health information, and testing of cognitive performance for those able to perform the tests, or proxy-reported information on cognitive ability for those unable to do the tests as well as those unwilling to answer for themselves.

We utilized 10 waves of HRS data spanning 2000 through 2018. Eligible people were those aged ≥65, having cognitive impairment (as defined below), and living alone.

The data are publicly available. The use of secondary deidentified data makes this study exempt from institutional review board review. This study follows the Strengthening the Reporting of Observational Studies in Epidemiology reporting guideline (20).

### Outcome and Measures

#### Individuals with cognitive impairment

Considering the potential for reversion of cognitive impairment (21), cognitive impairment was judged for each wave, and was identified by using a validated algorithm designed for HRS-based studies of dementia (12,13,22,23). The algorithm incorporates performance scores of Telephone Interview for Cognitive Status (TICS), and scores of proxy-reported information on cognitive impairment and functional limitations (proxy index). The TICS is a 27-point cognitive scale that included an immediate and delayed 10-noun free recall test, a serial 7s subtraction test, and a backwards-count-from-20 test. The proxy index is an 11-point scale, covering the participant’s memory, limitations in 5 instrumental activities of daily living (IADLs; defined below), and difficulty completing the interview because of a cognitive limitation. Participants were classified as having probable dementia if they scored 6 or lower on the TICS or scored 6 or more on the proxy index. Participants with cognitive impairment no dementia (CIND) were those who scored 7–11 on the TICS or 3–5 on the proxy index. Full details about the TICS and proxy assessment can be found elsewhere (12,13,22,23).

*Physical disability* includes disability identified from basic activities of daily living (BADLs) and IADLs. Participants with BADL disability were defined as those who reported difficulty in 1 or more of 6 BADL items (dressing, walking across a room, bathing, eating, getting in and out of bed, toileting). Participants with IADL disability were defined as those who reported difficulty in 1 or more of 5 IADL items (preparing a hot meal, shopping for groceries, making phone calls, taking medications, and managing money) (17,24,25). We distinguished BADL disability from IADL disability because disability in activities is developed in a progressive manner associated with cognitive decline (4). BADLs are related to basic activities that allow people to care for themselves, while IADLs are related to more complex activities that allow an individual to live independently in a community. The distinction between BADL and IADL disability can inform customized interventions to meet the needs of patients with physical disability (26).

*Social support* was assessed by questionnaire items corresponding to the 11 BADLs/IADLs listed above. For each item, respondents were asked if they received help from others. To gain insight into the social support received by respondents, we adopted 2 concepts used in the evaluation of health care utilization, namely a “contact process” (is support provided?) and a “frequency process” (how often or how much is support provided?) (27). In this study, to examine any unmet needs for social support, the contact process corresponded to 2 binary (yes/no) variables indicating whether respondents with physical disability received no BADL or (separately) no IADL support. We refer to someone as “BADL-unsupported” if they report some BADL disability but received no support for BADLs, and “IADL-unsupported” likewise. The frequency process corresponds to a counting variable indicating the number of unmet social support needs, assessed by calculating the difference between the number of BADL or IADL difficulties and the number of BADLs/IADLs for which some support was provided.

### Statistical Analysis

To describe the baseline characteristics, categorical variables were reported as number (percentage), and continuous variables were reported as mean (standard deviation, *SD*).

For binary outcomes, to estimate linear trends over time, we fitted logistic regression models by including year as the key predictor, controlling for age, gender, racial/ethnic status, whether a proxy response was required (yes vs no), and dementia status (probable CIND vs probable dementia; eqn 1).


$$\begin{array}{l} Logit(P)=\alpha +\beta *year+\gamma_{1}*x_{1}+\ldots +\gamma_{n}*x_{n}+\varepsilon \end{array}$$ (1)

where Logit(P) is the log odds of a binary outcome (such as reporting BADL disability); year is a continuous variable; $x_{1}+\ldots +x_{n}$ are the covariates controlled for. The odds ratio (OR) associated with “year” represents, for example, the change in the odds of BADL disability, per year; OR > 1 indicates an increasing quantity across the study period, and OR < 1 is the converse.

To estimate gender disparities in trends, we fitted a similar model but added the interaction between gender and year (eqn 2). We tested for racial/ethnic disparity similarly.


$$\begin{array}{l} Logit(P)=\alpha +\beta *year+\theta *year\\ \times gender+\gamma_{1}*x_{1}+\ldots +\gamma_{n}*x_{n}+\varepsilon \end{array}$$ (2)

Equivalent Poisson regressions were conducted for integer (counting) outcomes, but with the outcome variable as log(λ), where λ is the number of occurrences.

Survey weights were used to account for sampling design (including the unequal probability of selection, clustering, and stratification) and study attrition. The weight values were provided directly in the HRS data sets. Details of how the weights were calculated can be found elsewhere (28).

All analyses were completed using R, version 3.6.0. We report 2-tailed *p* values and 95% confidence intervals (CIs) throughout. *P* < .05 was considered to be statistically significant.

## Results

### Basic Description, Including BADL/IADL Impairment and Support

From the HRS 2000–2018, a total of 20 070 eligible respondents aged 65+ with cognitive impairment who lived alone were included in this study, including 12 466 (62.1%) respondents having probable CIND and 9 190 (45.8%) respondents having probable dementia. Table 1 summarizes their basic characteristics. Participants’ mean (*SD*) age was 80.9 (8.6) years, and the majority were women (75.4%) and White (59.5%).

**Table 1.** Basic description of the sample.

Variable	*N* (%)	Mean (*SD*)
Number (total)	20 070 (100.0)	
Age (years)		80.9 (8.6)
Gender (female)	15 123 (75.4)	
Race/ethnicity		
Hispanic	2 426 (12.1)	
Non-Hispanic Black	5 222 (26.0)	
Non-Hispanic other	475 (2.4)	
Non-Hispanic White	11 945 (59.5)	
Proxy response (yes)	4 766 (23.7)	
Physical disability		
BADL disability (yes)	9 596 (47.8)	
IADL disability (yes)	9 830 (49.0)	
Both BADL and IADL disability (yes)	7 543 (37.6)	
Whether in receipt of BADL/IADL social support, among those with corresponding disability		
BADL-unsupported (yes)	3 155 (32.9)	
IADL-unsupported (yes)	1 188 (12.1)	
Unmet BADL/IADL support needs, among those receiving BADL/IADL support		
Number of unmet BADL support needs		0.58 (0.9)
Number of unmet IADL support needs		0.98 (1.4)
Probable CIND or dementia	20 070 (100)	
Probable CIND (yes)	12 466 (62.1)	
Probable dementia (yes)	9 190 (45.8)	

*Notes*: BADL = basic activity of daily living; CIND = cognitive impairment no dementia; IADL = instrumental activity of daily living; *SD* = standard deviation.

Overall, 47.8% of eligible respondents reported some BADL disability, of whom 32.9% received no BADL support. Among those who received BADL support, the mean (*SD*) number of unmet BADL support needs was 0.58 (0.88).

Overall, 49% of the eligible respondents reported some IADL disability, of whom 12.1% received no IADL support. Among those who received IADL support, the mean (*SD*) number of unmet IADL support needs was 0.98 (1.35).

### Gender or Racial/Ethnic Differences in BADL/IADL Impairment

Females had a higher likelihood of reporting BADL disability (OR 1.43, CI 1.31–1.56) and IADL disability (OR 1.37, CI 1.25–1.49) compared with males (Table 2, Model 1). Compared with White respondents, Hispanic and Black respondents had a higher likelihood of reporting BADL disability (OR 1.45, CI 1.3–1.63; OR 1.22, CI 1.11–1.33, respectively) and IADL disability (OR 1.36, CI 1.22–1.53; OR 1.13, CI 1.03–1.24, respectively; Table 2, Model 1).

**Table 2.** Regression Analyses of Time Trends in the Prevalence of BADL or IADL Disability and Social Support.

Outcome	Variable	Model 1		Model 2		Model 3	
		OR (95% CI)	*p*	OR (95% CI)	*p*	OR (95% CI)	*p*
BADL disability	Year	1.00 (0.99, 1.01)	.8653	1.00 (0.98, 1.01)	.6703	0.99 (0.99, 1.00)	.1068
	Age	**1.04 (1.04, 1.05)**	**<.0001**	**1.04 (1.04, 1.05)**	**<.0001**	**1.04 (1.04, 1.05)**	**<.0001**
	Gender (female)	**1.43 (1.31, 1.56)**	**<.0001**	**1.39 (1.19, 1.62)**	**<.0001**	**1.43 (1.31, 1.56)**	**<.0001**
	Race/ethnicity (ref: non-Hispanic White)						
	Hispanic	**1.45 (1.3, 1.63)**	**<.0001**	**1.45 (1.30, 1.63)**	**<.0001**	1.13 (0.91, 1.39)	.265
	Non-Hispanic Black	**1.22 (1.11, 1.33)**	**<.0001**	**1.22 (1.11, 1.33)**	**<.0001**	1.02 (0.88, 1.19)	.8046
	Non-Hispanic other	1.06 (0.85, 1.33)	.5964	1.06 (0.85, 1.33)	.602	1.18 (0.79, 1.78)	.4146
	Proxy response (yes)	**3.03 (2.69, 3.41)**	**<.0001**	**3.03 (2.69, 3.42)**	**<.0001**	**3.03 (2.69, 3.42)**	**<.0001**
	Probable CIND vs dementia (dementia)	1.09 (1.0, 1.19)	.0622	1.08 (1.00, 1.19)	.0623	1.09(1.00, 1.20)	.0500
	Year × gender (female)			1.00 (0.99, 1.02)	.6846		
	Year × race/ethnicity (Hispanic)					**1.03 (1.01, 1.05)**	**.0119**
	Year × race/ethnicity (non-Hispanic Black)					**1.02 (1.00, 1.03)**	**.0158**
	Year × race/ethnicity (non-Hispanic other)					0.99 (0.95, 1.03)	.6065
IADL disability	Year	1.00 (0.99, 1.01)	.9453	1.00 (0.98, 1.01)	.6818	0.99 (0.99, 1.00)	.1545
	Age	**1.06 (1.05, 1.06)**	**<.0001**	**1.06 (1.05, 1.06)**	**<.0001**	**1.06 (1.05, 1.06)**	**<.0001**
	Gender (female)	**1.37 (1.25, 1.49)**	**<.0001**	**1.31 (1.13, 1.53)**	**.0005**	**1.36 (1.25, 1.49)**	**<.0001**
	Race/ethnicity (ref: non-Hispanic White)						
	Hispanic	**1.36 (1.22, 1.53)**	**<.0001**	**1.36 (1.21, 1.53)**	**<.0001**	0.96 (0.78, 1.18)	.7001
	Non-Hispanic Black	**1.13 (1.03, 1.24)**	**.0072**	**1.13 (1.03, 1.24)**	**.0072**	1.01 (0.87, 1.18)	.8559
	Non-Hispanic other	1.13 (0.90, 1.41)	.2904	1.13 (0.90, 1.41)	.2952	1.11 (0.74, 1.66)	.6121
	Proxy response (yes)	**4.62 (4.10, 5.26)**	**<.0001**	**4.62 (4.10, 5.26)**	**<.0001**	**4.66 (4.10, 5.31)**	**<.0001**
	Probable CIND vs dementia (dementia)	**1.46 (1.34, 1.60)**	**<.0001**	**1.46 (1.34, 1.60)**	**<.0001**	**1.48 (1.35, 1.62)**	**<.0001**
	Year × gender (female)			1.00 (0.99, 1.02)	.6016		
	Year × race/ethnicity (Hispanic)					**1.04 (1.01, 1.06)**	**.0007**
	Year × race/ethnicity (non-Hispanic Black)					1.01 (1.00, 1.03)	.1389
	Year × race/ethnicity (non-Hispanic other)					1.00 (0.96, 1.04)	.915
BADL-unsupported among those with BADL disability	Year	**0.98 (0.97, 0.99)**	**<.0001**	0.99 (0.97, 1.01)	.2761	**0.97 (0.96, 0.98)**	**<.0001**
	Age	**0.95 (0.94, 0.95)**	**<.0001**	**0.95 (0.94, 0.95)**	**<.0001**	**0.95 (0.94, 0.95)**	**<.0001**
	Gender (female)	**0.68 (0.59, 0.78)**	**<.0001**	0.78 (0.60, 1.00)	.052	**0.68 (0.59, 0.78)**	**<.0001**
	Race/ethnicity (ref: non-Hispanic White)						
	Hispanic	**0.57 (0.48, 0.67)**	**<.0001**	**0.57 (0.48, 0.68)**	**<.0001**	**0.57 (0.41, 0.79)**	**.0007**
	Non-Hispanic Black	**0.73 (0.63, 0.84)**	**<.0001**	**0.73 (0.63, 0.84)**	**<.0001**	**0.56 (0.44, 0.72)**	**<.0001**
	Non-Hispanic other	0.78 (0.55, 1.10)	.1609	0.79 (0.56, 1.11)	.173	0.56 (0.30, 1.03)	.0632
	Proxy response (yes)	**0.18 (0.15, 0.23)**	**<.0001**	**0.18 (0.15, 0.23)**	**<.0001**	**0.18 (0.15, 0.23)**	**<.0001**
	Probable CIND vs dementia (dementia)	**0.73 (0.64, 0.84)**	**<.0001**	**0.73 (0.64, 0.84)**	**<.0001**	**0.73 (0.64, 0.84)**	**<.0001**
	Year × gender (female)			0.99 (0.96, 1.01)	.3015		
	Year × race/ethnicity (Hispanic)					1.00 (0.97, 1.03)	.9659
	Year × race/ethnicity (non-Hispanic Black)					**1.03 (1.00, 1.05)**	**.0271**
	Year × race/ethnicity (non-Hispanic other)					1.04 (0.98, 1.10)	.2334
IADL-unsupported among those with IADL disability	Year	**1.02 (1.01, 1.04)**	**.0022**	**1.04 (1.02, 1.07)**	**.0023**	**1.02 (1.00, 1.04)**	**.0472**
	Age	**0.95 (0.94, 0.96)**	**<.0001**	**0.95 (0.94, 0.96)**	**<.0001**	**0.95 (0.94, 0.96)**	**<.0001**
	Gender (female)	**0.56 (0.46, 0.68)**	**<.0001**	0.99 (0.96, 1.01)	.3015	**0.56 (0.46, 0.68)**	**<.0001**
	Race/ethnicity (ref: non-Hispanic White)						
	Hispanic	**0.58 (0.44, 0.78)**	**.0002**	**0.59 (0.44, 0.78)**	**.0003**	0.68 (0.40, 1.13)	.1342
	Non-Hispanic Black	0.93 (0.75, 1.14)	.478	0.92 (0.75, 1.14)	.4543	0.76 (0.52, 1.09)	.1405
	Non-Hispanic other	0.99 (0.61, 1.61)	.9719	1.01 (0.62, 1.64)	.9697	0.68 (0.25, 1.82)	.4422
	Proxy response (yes)	**0.22 (0.15, 0.30)**	**<.0001**	**0.22 (0.16, 0.30)**	**<.0001**	**0.22 (0.15, 0.30)**	**<.0001**
	Probable CIND vs dementia (dementia)	**0.77 (0.63, 0.95)**	**.0141**	**0.77 (0.63, 0.95)**	**.0134**	**0.78 (0.63, 0.95)**	**.0155**
	Year × gender (female)			0.97 (0.94, 1.00)	.0753		
	Year × race/ethnicity (Hispanic)					0.99 (0.94, 1.03)	.5805
	Year × race/ethnicity (non-Hispanic Black)					1.02 (0.98, 1.06)	.2693
	Year × race/ethnicity (non-Hispanic other)					1.04 (0.95, 1.13)	.4125

*Notes*: BADL = basic activity of daily living; CIND = cognitive impairment no dementia; IADL = instrumental activity of daily living. Trends were measured by the adjusted odds ratio (OR) and its 95% confidence interval (CI), which was obtained from the coefficient of the “year” predictor in the logistic regression, controlling for age, gender, race/ethnicity, whether a proxy response was required, and dementia status. OR > 1 indicates an increasing trend in the quantity across the study years, and OR < 1 indicates a decreasing trend. Results were shown in boldface if their corresponding *p*-value < 0.05.

### BADL/IADL Impairment Over Time, With Gender or Racial/Ethnic Differences

**Figure 1.**
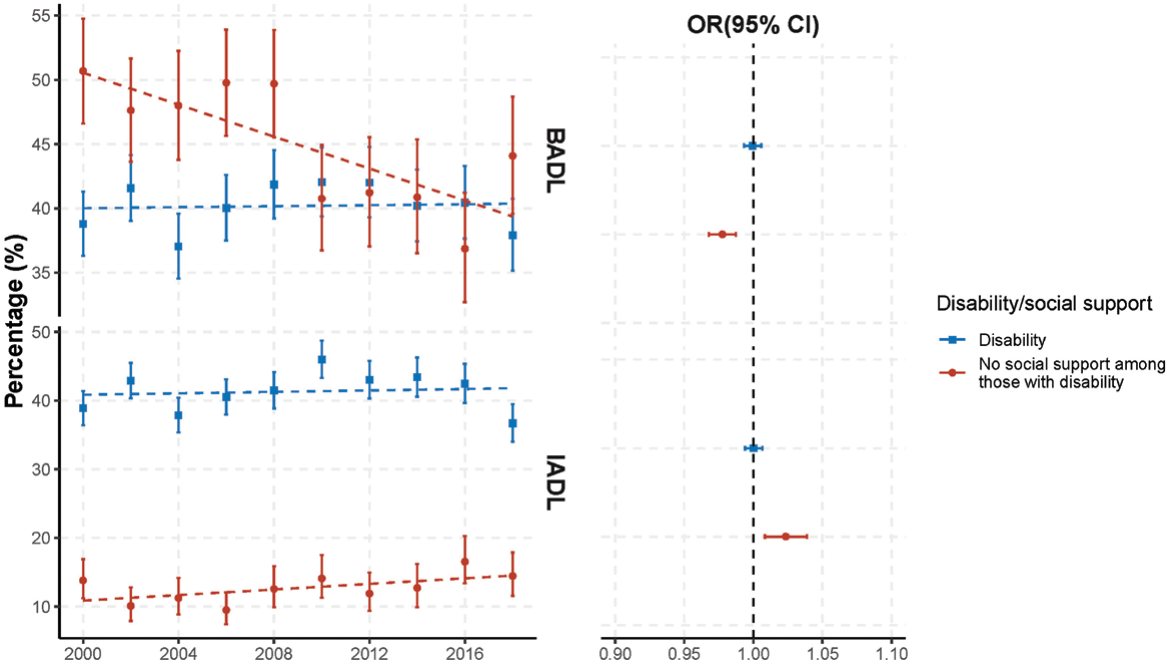
Time trends in the prevalence of BADL or IADL disability and social support among cognitively impaired older adults living alone in the United States, biennially from 2000 to 2018. BADL = basic activity of daily living; IADL = instrumental activity of daily living. The left panel presents the weighted percentage of BADL or IADL disability estimated from raw data, with error bars represent 95% confidence intervals (CIs). The dotted lines in the left panel show linear regression on the weighted percentage of BADL or IADL disability. The right panel shows the estimated time trend in the prevalence of BADL or IADL disability. Trends were measured via the adjusted odds ratio (OR) and its 95% CI, obtained from the coefficient of the “year” predictor in the logistic regression, controlling for age, gender, race/ethnicity, whether a proxy response was required, and dementia status. OR > 1 indicates an increasing trend in the prevalence across the study years, and OR < 1 indicates a decreasing trend.

From 2000 to 2018, no significant linear trends were found in the overall prevalence of BADL disability (OR 1.0, CI 0.99–1.01) or IADL disability (OR 1.0, CI 0.99–1.01; Table 2, Model 1; Figure 1). No gender disparities were found for these trends (Table 2, Model 2; Table 3, Model 2). Compared with White respondents, Hispanic and Black respondents had relatively increasing trends in BADL disability (OR 1.03, CI 1.01–1.05 and OR 1.02, CI 1.0–1.03, respectively; Table 2, Model 3). Hispanic respondents also had a relatively increasing trend in IADL disability (OR 1.04, CI 1.01–1.06; Table 2, Model 3).

**Table 3.** Regression Analyses of Time Trends in the Number of Unmet BADL or IADL Support Needs for Those Receiving BADL or IADL Support

Outcome	Variable	Model 1		Model 2		Model 3	
		RR (95% CI)	*p*	RR (95% CI)	*p*	RR (95% CI)	*p*
Number of unmet BADL support needs	Year	1.00 (0.99, 1.00)	.6037	1.00 (0.99, 1.01)	.7679	**0.99 (0.99, 1.00)**	**.0343**
	Age	**0.98 (0.97, 0.98)**	**<.0001**	**0.98 (0.97, 0.98)**	**<.0001**	**0.98 (0.97, 0.98)**	**<.0001**
	Gender (female)	0.96 (0.85, 1.08)	.5076	0.83 (0.66, 1.04)	.0986	0.95 (0.84, 1.07)	.4419
	Race/ethnicity (ref: non-Hispanic White)						
	Hispanic	1.00 (0.92, 1.08)	.9928	1.00 (0.92, 1.08)	.9946	**0.83 (0.70, 0.97)**	**.0185**
	Non-Hispanic Black	0.97 (0.90, 1.04)	.3913	0.97 (0.90, 1.04)	.3933	**0.86 (0.77, 0.96)**	**.0083**
	Non-Hispanic other	0.97 (0.83, 1.14)	.7279	0.97 (0.83, 1.15)	.7472	0.83 (0.63, 1.08)	.1736
	Proxy response (yes)	**0.51 (0.47, 0.56)**	**<.0001**	**0.51 (0.47, 0.56)**	**<.0001**	**0.51 (0.47, 0.56)**	**<.0001**
	Probable CIND vs dementia (dementia)	**0.93 (0.88, 0.99)**	**.0293**	**0.93 (0.88, 0.99)**	**.0294**	**0.93 (0.88, 0.99)**	**.0254**
	Year × gender (female)			1.00 (0.99, 1.01)	.5288		
	Year × race/ethnicity (Hispanic)					**1.02 (1.00, 1.03)**	**.0138**
	Year × race/ethnicity (non-Hispanic Black)					**1.01 (1.00, 1.02)**	**.0258**
	Year × race/ethnicity (non-Hispanic other)					1.02 (0.99, 1.04)	.189
Number of unmet IADL support needs	Year	**1.04 (1.03, 1.05)**	**<.0001**	**1.04 (1.03, 1.06)**	**<.0001**	**1.04 (1.03, 1.05)**	**<.0001**
	Age	**0.99 (0.98, 0.99)**	**<.0001**	**0.99 (0.98, 0.99)**	**<.0001**	**0.99 (0.98, 0.99)**	**.0001**
	Gender (female)	0.92 (0.83, 1.02)	.1127	0.92 (0.75, 1.14)	.4636	0.91 (0.83, 1.02)	.1025
	Race/ethnicity (ref: non-Hispanic White)						
	Hispanic	**0.61 (0.52, 0.71)**	**<.0001**	**0.61 (0.52, 0.71)**	**<.0001**	0.76 (0.56, 1.03)	.076
	Non-Hispanic Black	**0.77 (0.69, 0.87)**	**<.0001**	**0.77 (0.69, 0.87)**	**<.0001**	0.88 (0.70, 1.08)	.2222
	Non-Hispanic other	0.96 (0.71, 1.31)	.8163	0.96 (0.71, 1.31)	.8174	0.55 (0.27, 1.09)	.0874
	Proxy response (yes)	**1.35 (1.19, 1.54)**	**<.0001**	**1.35 (1.19, 1.54)**	**<.0001**	**1.35 (1.19, 1.54)**	**<.0001**
	Probable CIND vs dementia (dementia)	**0.84 (0.76, 0.94)**	**.0023**	**0.84 (0.76, 0.94)**	**.0023**	**0.84 (0.76, 0.94)**	**.0024**
	Year × gender (female)			1.00 (0.98, 1.02)	.9623		
	Year × race/ethnicity (Hispanic)					0.98 (0.96, 1.01)	.1299
	Year × race/ethnicity (non-Hispanic Black)					0.99 (0.97, 1.01)	.2455
	Year × race/ethnicity (non-Hispanic other)					1.05 (0.99, 1.11)	.1035

*Notes*: BADL = basic activity of daily living; CIND = cognitive impairment no dementia; IADL = instrumental activity of daily living. Trends were measured by the adjusted relative risk (RR) and its 95% confidence interval (CI), which was obtained from the coefficient of the “year” predictor in the Poisson regression, controlling for age, gender, race/ethnicity, whether a proxy response was required, and dementia status. RR > 1 indicates an increasing trend in the number of unmet BADL or IADL support needs across the study years, and RR < 1 indicates a decreasing trend. Results were shown in boldface if their corresponding *p*-value < 0.05.

### Gender or Racial/Ethnic Differences in BADL/IADL Support

Among those who reported disability, females were less likely to be BADL-unsupported (OR 0.68, CI 0.59–0.78) and IADL-unsupported (OR 0.56, CI 0.46–0.68), compared with males. Hispanic and Black respondents were less likely to be BADL-unsupported (OR 0.57, CI 0.48–0.67; OR 0.73, CI 0.63–0.84, respectively), compared with White respondents; and Hispanic respondents were also less likely to be IADL-unsupported (OR 0.58, CI 0.44–0.78; Table 2, Model 1).

Among those who reported disability and receipt of BADL/IADL support, no gender difference was found in the number of unmet BADL support needs (RR 0.96, CI 0.85–1.08) or unmet IADL support needs (RR 0.92, CI 0.83–1.02). Hispanic and Black respondents had no difference in the number of unmet BADL support needs (RR 1.00, CI 0.92–1.08 and RR 0.97, CI 0.90–1.04, respectively), but had significantly fewer unmet IADL support needs (RR 0.61, CI 0.52–0.71 and RR 0.77, CI 0.69–0.87, respectively), compared to White respondents (Table 3, Model 1).

### BADL/IADL Support Over Time, With Gender or Racial/Ethnic Differences

**Figure 2.**
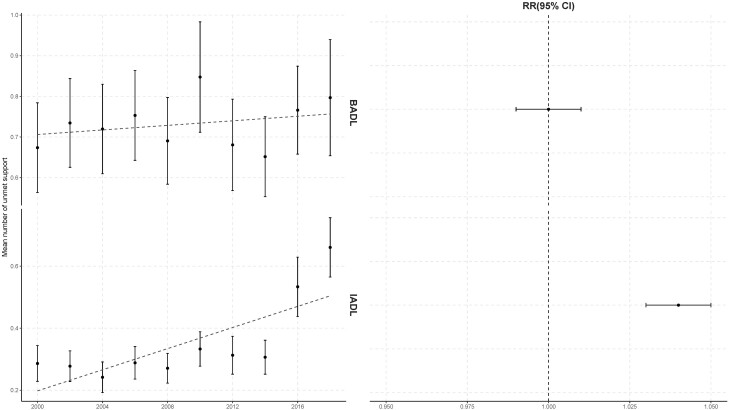
Time trends in the number of unmet BADL or IADL support needs among cognitively impaired adults living alone who were receiving BADL or IADL support, biennially from 2000 to 2018. BADL = basic activity of daily living; IADL = instrumental activity of daily living. The left panel presents the weighted mean number of unmet BADL or IADL support needs estimated from raw data, with error bars representing 95% confidence intervals (CIs). The dotted lines in the left panel show linear regression on the weighted mean number of unmet BADL or IADL support needs. The right panel shows the estimated time trend in the number of unmet BADL or IADL support needs. Trends were measured via the adjusted relative risk (RR) and its 95% CI, which was obtained from the coefficient of the “year” predictor in the Poisson regression, controlling for age, gender, race/ethnicity, whether a proxy response was required, and dementia status. RR > 1 indicates an increasing trend in the number of unmet BADL or IADL support needs across the study years, and RR < 1 is the converse.

The proportion of people unsupported for BADL needs decreased significantly over time (OR 0.98, CI 0.97–0.99), but the proportion of people unsupported for IADL needs increased (OR 1.02, CI 1.01–1.04; Table 2, Model 1; Figure 1). No significant trend was found in the number of unmet BADL support needs among those receiving BADL support (RR 1.00, CI 0.99–1.00), but among those receiving IADL support, the number of unmet IADL support needs increased over time (RR 1.04, CI 1.03–1.05; Table 3, Model 1; Figure 2).

No gender disparities were found for these trends (Table 2, Model 2; Table 3, Model 2). No racial/ethnic disparities were found in the trends for receipt of BADL or IADL support, except that Black respondents had a relatively increasing trend of being BADL-unsupported (OR 1.03, CI 1.0–1.05; Table 2, Model 3; Supplementary Figure 7) and Hispanic and Black respondents had a relatively increasing trend in the number of unmet BADL needs (RR 1.02, CI 1.00–1.03 and RR 1.01, CI 1.00–1.02, respectively; Table 3, Model 3; Supplementary Figure 8), compared to the corresponding trends in White respondents. Note, however, the overall differences discussed above: the relatively worse trend of a lesser reduction in support for BADL over time among Black respondents relative to White respondents was on the background of a better situation overall (that Black respondents, like Hispanic respondents, were overall more likely than White respondents to be supported—less likely to be unsupported—for BADL needs, discussed above), which is compatible with a slight narrowing of racial/ethnic disparity over time. For the number of unsupported BADL needs, there was greater deterioration over time among Hispanic/Black respondents than White respondents; for the number of IADL needs, there was an increase across racial/ethnic groups but a better situation (fewer unmet needs) for Black/Hispanic respondents independent of time.

### Subgroup by Cognitive Impairment No Dementia (CIND) and Dementia

Subgroup analyses (Supplementary Tables 1–4) indicated that the above racial/ethnic disparities in the trend of reporting BADL disability, being BADL-unsupported, and the number of unmet BADL support needs were mainly identified among those with dementia rather than CIND, while IADL-related disparities were identified among both people with CIND and dementia.

### Unmet Support Needs by Items of ADL and IADL

The proportions of respondents with unmet support needs are reported for each BADL/IADL item in Supplementary Figures 1–6. Compared to males, females reported more unmet support needs for toileting, walking, preparing a hot meal, and shopping for groceries; while compared to females, males had more unmet support needs for dressing (Supplementary Figure 1). Compared to White and Black respondents, Hispanic people reported more unmet needs for getting in/out of bed, dressing, and eating. Compared to White and Hispanic respondents, Black people reported more unmet needs for dressing, toileting, walking, preparing a hot meal, and shopping for groceries. Compared to Black and Hispanic respondents, White people reported more unmet needs for preparing a hot meal, taking medications, making phone calls, and shopping for groceries (Supplementary Figure 4). People with CIND had more unmet BADL support needs than unmet IADL support needs, while people with dementia had more unmet IADL support needs than unmet BADL support needs (Supplementary Figures 2–3 and 5–6).

## Discussion

### Statement of Principal Findings

This study assessed trends in BADL and IADL disability and social support among cognitively impaired U.S. older adults living alone, and the influence of gender and racial/ethnic disparities. Overall, between 2000 and 2018, the proportion of people who were BADL-unsupported decreased, while those who were IADL-unsupported increased. Females had a higher likelihood of reporting BADL and IADL disability compared to males. Hispanic and Black respondents had a higher likelihood of reporting BADL and IADL disability compared to White respondents. Among those who reported BADL or IADL disability, female, Hispanic, and Black respondents were more likely to be in receipt of BADL or IADL support. Among those receiving BADL or IADL support, there were no gender disparities in the number of unmet BADL or IADL support needs, and Hispanic and Black respondents had a lower number of unmet IADL support needs compared to White respondents.

Over time, fewer people with BADL disability reported being BADL-unsupported, but more respondents with IADL disability reported being IADL-unsupported, and among those who did receive IADL support, the number of unmet IADL support needs increased over time. There were no gender disparities in the trends in proportion of being BADL- or IADL-unsupported, or in number of unmet BADL or IADL support needs. Overall improvements in BADL support were seen over time, but less so in Black respondents. The number of unmet BADL needs increased more in Black and Hispanic respondents over time, relative to White respondents. Unmet support needs by specific BADL/IADL items were also reported (Supplementary Figures 1–6).

### Interpretation

Our study identified some gender disparities, including that females had a higher likelihood of suffering BADL and IADL disability compared to males. The results are consistent with another recent study that showed females were more likely to suffer from impairment in BADLs caused by cognitive impairment than males (17). Nevertheless, females were more likely to receive BADL or IADL support. This is consistent with other findings from the United States (29,30) and other countries (31), which indicated that females are more likely to receive social support than males. We also found that among those receiving BADL/IADL support, there were no gender disparities in the number of unmet BADL/IADL support needs. The above findings indicated that the gender disparity may be a result of difficulties in a “contact” rather than a “frequency” process (described below). Possible explanations might be that females are, on average, more active in neighborhood social networks and are more likely to ask for help or to contact other people, when in need (25,32). A customized intervention aiming at the contact process may be more effective in eliminating this gender disparity.

We identified racial/ethnic disparities in the prevalence of BADL and IADL disability, as well as the provision of corresponding social support. Black and Hispanic respondents were more likely to suffer from BADL/IADL disability than their White counterparts. This finding is in accordance with prior studies conducted in the United States that found Black and Hispanic people were at greater risk for dementia and functional disability (10–16). However, compared to White people, Black and Hispanic people were also more likely to receive BADL or IADL support, and had a lower number of unmet IADL needs. Racial/ethnic disparities were also identified in the time trends in the prevalence of BADL disability and corresponding receipt of BADL support. Given the baseline higher probability of reporting BADL disability among Hispanic and Black respondents than White, the relatively increasing trend identified in the prevalence of BADL disability among Hispanic and Black than White respondents revealed that an increasing number of Hispanic and Black respondents reported BADL disability over time. Compared to the corresponding trends in White respondents, we also identified a relatively increasing trend of being BADL-unsupported among Black respondents, but no such difference was identified among Hispanic respondents. Given the baseline difference of a lower likelihood of being BADL-unsupported among Hispanic and Black communities than White, these differences in the time trends of being BADL-unsupported are compatible with some narrowing of disparity over time. We display these trends in Supplementary Figure 7, showing that they resulted from an improvement in the receipt of BADL support among White and Hispanic communities while there was almost no improvement among the Black community. Similarly, Supplementary Figure 8 indicates that the relatively increasing trend in the number of unmet BADL support needs among Hispanic and Black (vs White) respondents was primarily because that Hispanic and Black respondents have been facing increasing numbers of unmet BADL support needs over time. These findings indicate that from 2000 to 2018, ethnic minorities with cognitive impairment living alone had greater or unimproved unmet needs for BADL support, both in terms of a “contact” process (Black community) and a “frequency” process (Hispanic and Black communities). A customized intervention targeting at the different processes for different racial/ethnic communities may be more effective in eliminating this disparity.

Possible reasons for the change in the above racial/ethnic disparities could include the entanglement of potential risk factors, protective factors, and resilience among racial/ethnic groups. Ethnic minorities were more likely to be exposed to high occupational risks and thus had a higher probability of suffering disability in older age (33). With informal care (mainly from families and friends) being the primary source of support for U.S. older adults with cognitive impairment (1), studies have found that people from ethnic minorities were more likely to devote time to informal care than those of White ethnicity. According to a caregiving report in the United States, caregivers of ethnic minorities report providing more hours of care, on average, to their older recipients than White caregivers and are more likely to provide 21 or more hours of care weekly (34). Extensive costs for long-term care have been a challenge to those in need to access to formal care (35). Medicaid programs in many states have expanded home care and shifted funds toward home- and community-based services in recent decades, which could also facilitate some ethnic minorities to benefit from this expansion. However, the shortages in the long-term care workforce may disproportionately allocate a limited workforce to those covered by private long-term care insurance, where White Americans may have some advantages in terms of affordability (36,37).

Our subgroup analyses on CIND and dementia indicated that these BADL-related racial/ethnic disparities mainly occurred in people with dementia but not those with CIND. This difference between people with dementia and those with CIND is to some extent in keeping with recent research showing that caregivers for an adult aged ≥50 years with Alzheimer’s disease are more likely to have difficulties assisting their recipients with BADLs than those who provide care to someone without Alzheimer’s disease (34). Intervention programs could be targeted and used to narrow these racial/ethnic disparities in the unmet BADL-related needs, especially in vulnerable subgroups with dementia.

As for the receipt of IADL social support, no corresponding gender or racial/ethnic disparities were found, but more people with IADL disabilities faced unmet IADL support needs across the period 2000–2018. This was observed both in the “contact” process (do people in need receive some sort of care?) and the “frequency” process (when in receipt of help, does this meet the need?). In particular, there were indications that number of unmet IADL support needs has increased more sharply recent years (Figure 2). Further, our subgroup analyses on CIND and dementia indicated that among people with CIND, the above unmet IADL support manifested mostly in support “frequency” (received support does not meet the need), while among people with dementia, the IADL needs were less well met both in terms of contact (cannot connect with supporter) and frequency. This highlights the potential necessity of customized interventions for people with CIND and dementia separately.

We also found that unmet support needs exhibited obvious variation between gender, race/ethnicity, and people with CIND or dementia (eg, females reported more unmet support needs for toileting, walking, preparing a hot meal, and shopping for groceries, while males reported more unmet support needs for dressing). This variation might come from people’s personalities (how well they get along with outsiders), their acceptance of personal services (especially services involving personal privacy), and the type of service personnel (formal or informal) (4). In practice, this variation suggests that it is necessary to provide targeted and personalized services for specific service objects. For instance, mobility equipment and devices tailored to individual needs and circumstances might substitute for human assistance and facilitate self-care for some daily activities (38). Adequate provision of home- and community-based services, such as home-visit medical services, self-help support groups, and respite care, could also reduce unmet needs among vulnerable subgroups with dementia (39). Furthermore, the needs of people with cognitive impairment are complex and coordination between different agencies in the health and social care systems is not always efficient, leading to inadequate measures of unmet need among this population (1,40). Therefore, to ensure the integrity of services provided to people with cognitive impairment, it is important to assess needs regularly and determine what types of services, or combination of service types, are required.

### Strength and Limitations

To our knowledge, this is the first study to assess systematically the influence of gender and race/ethnicity on disabilities relating to ADL, and social support for them, among U.S. older adults living alone with cognitive impairment using population-based and nationally representative data. One strength of our study is that we give separate attention to BADL and IADL; the former is related to basic activities and the latter is related to more complex activities (26). Another strength is that we divided the process of receiving social support into “contact” and “frequency” processes. Further, we reported unmet support needs by individual BADL/IADL items. All of these contribute to our understanding of how any gender and racial/ethnic disparities may arise, and provide detailed evidence to more nuanced and practical public health policy strategies.

A key study limitation is the lack of clinical diagnosis of cognitive impairment or dementia. However, prior validation studies showed at least 91% concordance for dementia when using algorithm adopted above compared with the detailed Aging, Demographics, and Memory Study clinical evaluation (23). Another limitation is an inevitable potential for bias resulting from self-reported and proxy-reported outcomes of disability and social support, as either might under- or overestimate difficulty or support received; however, both measures have also been validated previously (41). Thirdly, for our measure of unmet social support, we used the difference between the number of BADL or IADL difficulties and the number of items for which support was received. However, this may underestimate unmet needs, as the underlying hypothesis for this measurement is that each item of support people received completely met their corresponding need (whereas, eg, receiving some support for making phone calls may not imply that all such needs are met in practice). Fourthly, some people who receive certain support might not suggest that they need such support. Thus, when we explore the association between overall disability and overall social support, it is possible that the disability items may not correspond with the support items. This will also underestimate unmet needs.

One unanswered question is the interaction between gender and race/ethnicity. Although this study identified a higher likelihood for Hispanic and Black people to receive BADL or IADL support, a recent study showed that Black women were less likely to receive BADL/IADL support than comparable White women, whereas this difference in the outcome was not significant in men (17). A future study is needed with a focus on the interaction between gender and race/ethnicity.

## Conclusion

Among U.S. older adults with cognitive impairment living alone, although the overall prevalence of BADL and IADL disability remained steady between 2000 and 2018, fewer people received IADL support and the extent of unmet IADL support needs increased, over time. Gender disparities were seen in the prevalence of BADL or IADL disability, and lack of corresponding support, while racial/ethnic disparities were seen both in the prevalence of reported BADL/IADL disability and unmet needs for BADL/IADL support. Some racial/ethnic disparities narrowed, for example, with unmet BADL support needs worsening over time in minority ethnicity groups (Hispanic and Black) relative to the majority (White) ethnic group but from a better baseline. Data such as these allow for identifying groups most in need, and therefore the potential to target support interventions to have the greatest impact.

## Supplementary Material

igad028_suppl_Supplementary_Material

## Data Availability

Data are publicly available and can be accessed in https://www.rand.org/well-being/social-and-behavioral-policy/centers/aging/dataprod/hrs-data.html.
