# Supplementary material for: Trends in Gender and Racial/Ethnic Disparities in Physical Disability and Social Support Among U.S. Older Adults With Cognitive Impairment Living Alone, 2000–2018
Source: Innov Aging. 2023 Mar 21;7(4):igad028. doi: 10.1093/geroni/igad028 (PMC10202553; doi:10.1093/geroni/igad028)
Supplement: igad028_suppl_Supplementary_Material [file igad028_suppl_supplementary_material.docx]

**Supplementary Table 1. Regression analyses of time trends in the prevalence of BADL/IADL disability and social support (among those with CIND living alone).**

| **Outcome** | **Variable** | **Model 1** | | **Model 2** | | **Model 3** | |
| --- | --- | --- | --- | --- | --- | --- | --- |
|  |  | **OR (95%CI)** | ***p*** | **OR (95%CI)** | ***p*** | **OR (95%CI)** | ***p*** |
| BADL disability | Year | 1.01 (1.00, 1.01) | 0.1439 | 1.01 (0.99, 1.02) | 0.5052 | 1.00 (0.99, 1.01) | 0.6505 |
|  | Age | **1.02 (1.02, 1.03)** | **<0.0001** | **1.02 (1.02, 1.03)** | **<0.0001** | **1.02 (1.02, 1.03)** | **<0.0001** |
|  | Gender (female) | **1.43 (1.28, 1.62)** | **<0.0001** | **1.43 (1.16, 1.77)** | **0.0006** | **1.43 (1.28, 1.62)** | **<0.0001** |
|  | Race/ethnicity (non-Hispanic White) |  |  |  |  |  |  |
|  | Hispanic | **1.36 (1.17, 1.60)** | **0.0001** | **1.36 (1.17, 1.60)** | **0.0001** | 1.23 (0.93, 1.65) | 0.141 |
|  | non-Hispanic Black | **1.19 (1.05, 1.35)** | **0.0057** | **1.19 (1.05, 1.35)** | **0.0057** | 1.04 (0.84, 1.27) | 0.7428 |
|  | non-Hispanic other | 1.17 (0.86, 1.62) | 0.3064 | 1.17 (0.86, 1.62) | 0.3066 | 1.15 (0.63, 2.12) | 0.6551 |
|  | Year × gender (female) |  |  | 1.00 (0.98, 1.02) | 0.9931 |  |  |
|  | Year × race/ethnicity (Hispanic) |  |  |  |  | 1.01 (0.98, 1.04) | 0.4516 |
|  | Year × race/ethnicity (non-Hispanic Black) |  |  |  |  | 1.01 (0.99, 1.03) | 0.1768 |
|  | Year × race/ethnicity (non-Hispanic other) |  |  |  |  | 1.00 (0.95, 1.06) | 0.9208 |
| IADL disability | Year | **1.01 (1.00, 1.02)** | **0.0022** | 1.01 (0.99, 1.03) | 0.2842 | 1.00 (0.99, 1.02) | 0.3483 |
|  | Age | **1.04 (1.03, 1.04)** | **<0.0001** | **1.04 (1.03, 1.04)** | **<0.0001** | **1.04 (1.03, 1.04)** | **<0.0001** |
|  | Gender (female) | **1.35 (1.20, 1.52)** | **<0.0001** | **1.30 (1.05, 1.62)** | **0.016** | **1.35 (1.20, 1.52)** | **<0.0001** |
|  | Race/ethnicity (non-Hispanic White) |  |  |  |  |  |  |
|  | Hispanic | **1.20 (1.02, 1.40)** | **0.0261** | **1.20 (1.02, 1.40)** | **0.0265** | 0.80 (0.59, 1.08) | 0.1531 |
|  | non-Hispanic Black | 1.03 (0.90, 1.17) | 0.6643 | 1.03 (0.90, 1.17) | 0.6624 | 0.85 (0.68, 1.06) | 0.1518 |
|  | non-Hispanic other | 0.89 (0.64, 1.22) | 0.4594 | 0.89 (0.64, 1.22) | 0.4537 | 0.59 (0.30, 1.19) | 0.142 |
|  | Year × gender (female) |  |  | 1.00 (0.98, 1.02) | 0.712 |  |  |
|  | Year × race/ethnicity (Hispanic) |  |  |  |  | **1.04 (1.01, 1.07)** | **0.0058** |
|  | Year × race/ethnicity (non-Hispanic Black) |  |  |  |  | 1.02 (1.00, 1.04) | 0.0771 |
|  | Year × race/ethnicity (non-Hispanic other) |  |  |  |  | 1.04 (0.98, 1.11) | 0.2297 |
| BADL disability without receipt of corresponding support | Year | **0.96 (0.95, 0.97)** | **<0.0001** | **0.95 (0.91, 0.98)** | **0.0039** | **0.96 (0.94, 0.98)** | **<0.0001** |
|  | Age | **0.97 (0.96, 0.98)** | **<0.0001** | **0.97 (0.96, 0.98)** | **<0.0001** | **0.97 (0.96, 0.98)** | **<0.0001** |
|  | Gender (female) | **0.66 (0.53, 0.81)** | **0.0001** | **0.57 (0.38, 0.84)** | **0.0053** | **0.66 (0.54, 0.81)** | **0.0001** |
|  | Race/ethnicity (non-Hispanic White) |  |  |  |  |  |  |
|  | Hispanic | **0.58 (0.45, 0.76)** | **<0.0001** | **0.58 (0.45, 0.75)** | **<0.0001** | 0.69 (0.44, 1.09) | 0.114 |
|  | non-Hispanic Black | **0.76 (0.62, 0.93)** | **0.0094** | **0.76 (0.62, 0.93)** | **0.0091** | 0.70 (0.49, 1.01) | 0.0541 |
|  | non-Hispanic other | 0.97 (0.61, 1.54) | 0.8846 | 0.96 (0.60, 1.54) | 0.8691 | 1.28 (0.46, 3.53) | 0.6331 |
|  | Year × gender (female) |  |  | 1.01 (0.98, 1.05) | 0.4717 |  |  |
|  | Year × race/ethnicity (Hispanic) |  |  |  |  | 0.98 (0.94, 1.03) | 0.4834 |
|  | Year × race/ethnicity (non-Hispanic Black) |  |  |  |  | 1.01 (0.97, 1.04) | 0.6872 |
|  | Year × race/ethnicity (non-Hispanic other) |  |  |  |  | 0.97 (0.90, 1.06) | 0.5729 |
| IADL disability without receipt of corresponding support | Year | 1.01 (0.99, 1.03) | 0.4789 | 1.02 (0.99, 1.06) | 0.216 | 1.01 (0.98, 1.03) | 0.6307 |
|  | Age | **0.97 (0.96, 0.99)** | **0.0001** | **0.97 (0.96, 0.99)** | **0.0001** | **0.97 (0.96, 0.99)** | **0.0001** |
|  | Gender (female) | **0.56 (0.44, 0.71)** | **<0.0001** | 0.71 (0.45, 1.13) | 0.1491 | **0.56 (0.44, 0.71)** | **<0.0001** |
|  | Race/ethnicity (non-Hispanic White) |  |  |  |  |  |  |
|  | Hispanic | 0.79 (0.55, 1.13) | 0.1904 | 0.79 (0.55, 1.14) | 0.2033 | 0.76 (0.39, 1.49) | 0.4334 |
|  | non-Hispanic Black | 1.11 (0.83, 1.46) | 0.5042 | 1.09 (0.83, 1.45) | 0.5201 | 1.02 (0.63, 1.67) | 0.937 |
|  | non-Hispanic other | 1.20 (0.62, 2.32) | 0.6003 | 1.21 (0.63, 2.34) | 0.5687 | 1.55 (0.36, 6.82) | 0.5564 |
|  | Year × gender (female) |  |  | 0.98 (0.93, 1.02) | 0.2722 |  |  |
|  | Year × race/ethnicity (Hispanic) |  |  |  |  | 1.00 (0.94, 1.06) | 0.9345 |
|  | Year × race/ethnicity (non-Hispanic Black) |  |  |  |  | 1.01 (0.96, 1.05) | 0.7639 |
|  | Year × race/ethnicity (non-Hispanic other) |  |  |  |  | 0.98 (0.86, 1.11) | 0.7252 |

Notes. BADL=basic activity of daily living; IADL=instrumental activity of daily living; CIND=cognitive impairment, no dementia. Trends were measured by the adjusted odds ratio (OR) and its 95% confidence interval (CI), which was obtained from the coefficient of the “year” predictor in the logistic regression, controlling for age, gender, and race/ethnicity. Whether need proxy response was not controlled, as all people with probable CIND response by themselves. OR>1 indicates an increasing trend in the prevalence across the study years, and OR<1 the converse.

**Supplementary Table 2. Regression analyses of time trends in the prevalence of BADL/IADL disability and social support (among those with dementia living alone).**

| **Outcome** | **Variable** | **Model 1** | | **Model 2** | | **Model 3** | |
| --- | --- | --- | --- | --- | --- | --- | --- |
|  |  | **OR (95%CI)** | ***p*** | **OR (95%CI)** | ***p*** | **OR (95%CI)** | ***p*** |
| BADL disability | Year | **0.99 (0.98, 1.00)** | **0.01** | 0.98 (0.97, 1.00) | 0.1294 | **0.98 (0.96, 0.99)** | **0.0002** |
|  | Age | **1.06 (1.05, 1.06)** | **<0.0001** | **1.06 (1.05, 1.06)** | **<0.0001** | **1.06 (1.05, 1.06)** | **<0.0001** |
|  | Gender (female) | **1.46 (1.28, 1.68)** | **<0.0001** | **1.42 (1.13, 1.80)** | **0.0032** | **1.46 (1.28, 1.68)** | **<0.0001** |
|  | Race/ethnicity (non-Hispanic White) |  |  |  |  |  |  |
|  | Hispanic | **1.31 (1.09, 1.55)** | **0.0024** | **1.30 (1.09, 1.55)** | **0.0025** | 0.80 (0.58, 1.09) | 0.1634 |
|  | non-Hispanic Black | 1.04 (0.91, 1.19) | 0.5602 | 1.04 (0.91, 1.19) | 0.5598 | **0.78 (0.62, 0.98)** | **0.0303** |
|  | non-Hispanic other | 0.77 (0.55, 1.07) | 0.1217 | 0.77 (0.55, 1.07) | 0.1207 | 0.90 (0.52, 1.58) | 0.7321 |
|  | Proxy response (yes) | **2.92 (2.59, 3.29)** | **<0.0001** | **2.92 (2.59, 3.29)** | **<0.0001** | **2.94 (2.61, 3.32)** | **<0.0001** |
|  | Year × gender (female) |  |  | 1.00 (0.98, 1.03) | 0.7815 |  |  |
|  | Year × race/ethnicity (Hispanic) |  |  |  |  | **1.05 (1.02, 1.08)** | **0.0017** |
|  | Year × race/ethnicity (non-Hispanic Black) |  |  |  |  | **1.03 (1.01, 1.05)** | **0.0066** |
|  | Year × race/ethnicity (non-Hispanic other) |  |  |  |  | 0.98 (0.92, 1.04) | 0.5144 |
| IADL disability | Year | **0.98 (0.97, 0.99)** | **<0.0001** | 0.98 (0.96, 1.00) | 0.0635 | **0.97 (0.96, 0.98)** | **<0.0001** |
|  | Age | **1.07 (1.06, 1.08)** | **<0.0001** | **1.07 (1.06, 1.08)** | **<0.0001** | **1.07 (1.06, 1.08)** | **<0.0001** |
|  | Gender (female) | **1.51 (1.32, 1.73)** | **<0.0001** | **1.57 (1.23, 1.97)** | **0.0003** | **1.51 (1.32, 1.73)** | **<0.0001** |
|  | Race/ethnicity (non-Hispanic White) |  |  |  |  |  |  |
|  | Hispanic | **1.20 (1.01, 1.42)** | **0.042** | **1.20 (1.01, 1.43)** | **0.0414** | 0.81 (0.58, 1.13) | 0.2096 |
|  | non-Hispanic Black | 0.96 (0.84, 1.11) | 0.6061 | 0.96 (0.84, 1.11) | 0.6053 | 0.79 (0.63, 1.00) | 0.0524 |
|  | non-Hispanic other | 1.12 (0.80, 1.55) | 0.5032 | 1.12 (0.80, 1.57) | 0.5015 | 1.00 (0.57, 1.77) | 0.9979 |
|  | Proxy response (yes) | **4.48 (3.94, 5.10)** | **<0.0001** | **4.48 (3.94, 5.10)** | **<0.0001** | **4.53 (3.97, 5.16)** | **<0.0001** |
|  | Year × gender (female) |  |  | 1.00 (0.97, 1.02) | 0.7811 |  |  |
|  | Year × race/ethnicity (Hispanic) |  |  |  |  | **1.04 (1.01, 1.07)** | **0.0148** |
|  | Year × race/ethnicity (non-Hispanic Black) |  |  |  |  | 1.02 (1.00, 1.04) | 0.0728 |
|  | Year × race/ethnicity (non-Hispanic other) |  |  |  |  | 1.01 (0.96, 1.07) | 0.6824 |
| BADL disability without receipt of corresponding support | Year | 0.99 (0.98, 1.01) | 0.3972 | 1.02 (0.99, 1.05) | 0.2445 | **0.98 (0.96, 1.00)** | **0.0444** |
|  | Age | **0.94 (0.93, 0.95)** | **<0.0001** | **0.94 (0.93, 0.95)** | **<0.0001** | **0.94 (0.93, 0.95)** | **<0.0001** |
|  | Gender (female) | **0.63 (0.52, 0.78)** | **<0.0001** | 0.87 (0.59, 1.26) | 0.4551 | **0.63 (0.51, 0.77)** | **<0.0001** |
|  | Race/ethnicity (non-Hispanic White) |  |  |  |  |  |  |
|  | Hispanic | **0.70 (0.54, 0.90)** | **0.0061** | **0.70 (0.54, 0.90)** | **0.0071** | **0.52 (0.32, 0.86)** | **0.0113** |
|  | non-Hispanic Black | 0.87 (0.71, 1.06) | 0.1774 | 0.87 (0.71, 1.06) | 0.1696 | **0.58 (0.40, 0.83)** | **0.0029** |
|  | non-Hispanic other | 0.72 (0.42, 1.22) | 0.2258 | 0.74 (0.44, 1.26) | 0.2615 | **0.43 (0.19, 0.99)** | **0.048** |
|  | Proxy response (yes) | **0.19 (0.16, 0.23)** | **<0.0001** | **0.19 (0.16, 0.23)** | **<0.0001** | **0.19 (0.16, 0.23)** | **<0.0001** |
|  | Year × gender (female) |  |  | 0.97 (0.93, 1.00) | 0.0653 |  |  |
|  | Year × race/ethnicity (Hispanic) |  |  |  |  | 1.03 (0.98, 1.07) | 0.223 |
|  | Year × race/ethnicity (non-Hispanic Black) |  |  |  |  | **1.04 (1.01, 1.08)** | **0.0111** |
|  | Year × race/ethnicity (non-Hispanic other) |  |  |  |  | 1.06 (0.98, 1.15) | 0.159 |
| IADL disability without receipt of corresponding support | Year | **1.05 (1.02, 1.07)** | **0.0004** | **1.07 (1.03, 1.12)** | **0.0016** | **1.04 (1.01, 1.08)** | **0.0112** |
|  | Age | **0.93 (0.91, 0.94)** | **<0.0001** | **0.93 (0.91, 0.94)** | **<0.0001** | **0.93 (0.91, 0.95)** | **<0.0001** |
|  | Gender (female) | **0.51 (0.37, 0.69)** | **<0.0001** | 0.73 (0.41, 1.32) | 0.2976 | **0.51 (0.37, 0.70)** | **<0.0001** |
|  | Race/ethnicity (non-Hispanic White) |  |  |  |  |  |  |
|  | Hispanic | **0.47 (0.28, 0.78)** | **0.0038** | **0.47 (0.28, 0.79)** | **0.0043** | 0.81 (0.36, 1.84) | 0.6196 |
|  | non-Hispanic Black | 0.92 (0.66, 1.28) | 0.6355 | 0.92 (0.66, 1.27) | 0.6133 | 0.73 (0.41, 1.34) | 0.3117 |
|  | non-Hispanic other | 1.09 (0.51, 2.32) | 0.8184 | 1.11 (0.52, 2.36) | 0.7848 | 0.58 (0.15, 2.16) | 0.4167 |
|  | Proxy response (yes) | **0.23 (0.16, 0.32)** | **<0.0001** | **0.23 (0.16, 0.32)** | **<0.0001** | **0.22 (0.16, 0.31)** | **<0.0001** |
|  | Year × gender (female) |  |  | 0.97 (0.91, 1.02) | 0.193 |  |  |
|  | Year × race/ethnicity (Hispanic) |  |  |  |  | 0.95 (0.88, 1.02) | 0.1602 |
|  | Year × race/ethnicity (non-Hispanic Black) |  |  |  |  | 1.02 (0.97, 1.08) | 0.4194 |
|  | Year × race/ethnicity (non-Hispanic other) |  |  |  |  | 1.06 (0.94, 1.20) | 0.3109 |

Notes. BADL=basic activity of daily living; IADL=instrumental activity of daily living. Trends were measured by the adjusted odds ratio (OR) and its 95% confidence interval (CI), which was obtained from the coefficient of the “year” predictor in the logistic regression, controlling for age, gender, race/ethnicity, and whether need proxy response. OR>1 indicates an increasing trend in the prevalence across the study years, and OR<1 the converse.

**Supplementary Table 3. Regression analyses of time trends in the number of unmet BADL/IADL support needs for those receipt of BADL/IADL support (among those with CIND living alone).**

| **Outcome** | **Variable** | **Model 1** | | **Model 2** | | **Model 3** | |
| --- | --- | --- | --- | --- | --- | --- | --- |
|  |  | **RR (95% CI)** | ***p*** | **RR (95% CI)** | ***p*** | **RR (95% CI)** | ***p*** |
| Number of unmet BADL support needs | Year | 1.00 (0.99, 1.00) | 0.6895 | 1.01 (1.00, 1.02) | 0.2226 | 1.00 (0.99, 1.00) | 0.292 |
|  | Age | **0.99 (0.98, 0.99)** | **<0.0001** | **0.99 (0.98, 0.99)** | **<0.0001** | **0.99 (0.98, 0.99)** | **<0.0001** |
|  | Gender (female) | 0.94 (0.86, 1.02) | 0.1351 | 1.06 (0.91, 1.22) | 0.4388 | 0.93 (0.86, 1.02) | 0.1258 |
|  | Race/ethnicity (Ref: non-Hispanic White) |  |  |  |  |  |  |
|  | Hispanic | 1.04 (0.92, 1.16) | 0.5312 | 1.04 (0.92, 1.16) | 0.5133 | 0.90 (0.73, 1.11) | 0.3056 |
|  | non-Hispanic Black | 1.01 (0.92, 1.11) | 0.7777 | 1.01 (0.92, 1.11) | 0.7645 | 0.97 (0.85, 1.12) | 0.672 |
|  | non-Hispanic Other | 1.13 (0.94, 1.34) | 0.1926 | 1.13 (0.95, 1.35) | 0.1732 | 1.01 (0.76, 1.34) | 0.9716 |
|  | Year × gender (female) |  |  | 0.99 (0.97, 1.00) | 0.1049 |  |  |
|  | Year × race/ethnicity (Hispanic) |  |  |  |  | 1.01 (0.99, 1.03) | 0.1709 |
|  | Year × race/ethnicity (non-Hispanic Black) |  |  |  |  | 1.00 (0.99, 1.02) | 0.5325 |
|  | Year × race/ethnicity (non-Hispanic other) |  |  |  |  | 1.01 (0.98, 1.04) | 0.4484 |
| Number of unmet IADL support needs | Year | **1.02 (1.01, 1.03)** | **0.0022** | **1.02 (1.00, 1.04)** | **0.0164** | 1.01 (1.00, 1.02) | 0.0953 |
|  | Age | **0.98 (0.97, 0.99)** | **<0.0001** | **0.98 (0.97, 0.99)** | **<0.0001** | **0.98 (0.97, 0.99)** | **<0.0001** |
|  | Gender (female) | 0.92 (0.79, 1.06) | 0.2665 | 1.03 (0.79, 1.35) | 0.7987 | 0.91 (0.79, 1.06) | 0.241 |
|  | Race/ethnicity (Ref: non-Hispanic White) |  |  |  |  |  |  |
|  | Hispanic | **0.79 (0.65, 0.97)** | **0.025** | **0.79 (0.65, 0.97)** | **0.0273** | 0.73 (0.50, 1.08) | 0.1177 |
|  | non-Hispanic Black | **0.84 (0.70, 1.00)** | **0.0471** | **0.84 (0.70, 1.00)** | **0.0451** | **0.73 (0.55, 0.98)** | **0.0359** |
|  | non-Hispanic Other | 1.17 (0.72, 1.92) | 0.5305 | 1.17 (0.72, 1.92) | 0.5149 | **0.25 (0.07, 0.94)** | **0.0404** |
|  | Year × gender (female) |  |  | 0.99 (0.97, 1.01) | 0.3529 |  |  |
|  | Year × race/ethnicity (Hispanic) |  |  |  |  | 1.01 (0.98, 1.04) | 0.6049 |
|  | Year × race/ethnicity (non-Hispanic Black) |  |  |  |  | 1.01 (0.99, 1.04) | 0.3206 |
|  | Year × race/ethnicity (non-Hispanic other) |  |  |  |  | **1.13 (1.02, 1.23)** | **0.0204** |

Notes. BADL=basic activity of daily living; IADL=instrumental activity of daily living; CIND=cognitive impairment, no dementia. Trends were measured by the adjusted relative ratio (RR) and its 95% confidence interval (CI), which was obtained from the coefficient of the “year” predictor in the Poisson regression, controlling for age, gender, and race/ethnicity. Whether need proxy response was not controlled, as all people with probable CIND response by themselves. RR>1 indicates an increasing trend in the number of unmet BADL or IADL support needs across the study years, and RR<1 the converse. BADL, basic activity of daily living.

**Supplementary Table 4. Regression analyses of time trends in the number of unmet BADL or IADL support needs for those receipt of BADL or IADL support (among those with dementia living alone).**

| **Outcome** | **Variable** | **Model 1** | | **Model 2** | | **Model 3** | |
| --- | --- | --- | --- | --- | --- | --- | --- |
|  |  | **RR (95%CI)** | ***p*** | **RR (95%CI)** | ***p*** | **RR (95%CI)** | ***p*** |
| Number of unmet BADL support needs | Year | 1.00 (0.99, 1.01) | 0.5655 | 0.99 (0.98, 1.00) | 0.1365 | **0.99 (0.98, 1.00)** | **0.0476** |
|  | Age | **0.97 (0.97, 0.98)** | **<0.0001** | **0.97 (0.97, 0.98)** | **<0.0001** | **0.97 (0.97, 0.98)** | **<0.0001** |
|  | Gender (female) | **0.87 (0.79, 0.96)** | **0.0061** | **0.78 (0.65, 0.94)** | **0.0086** | **0.87 (0.79, 0.96)** | **0.0059** |
|  | Race/ethnicity (ref: non-Hispanic White) |  |  |  |  |  |  |
|  | Hispanic | 1.06 (0.94, 1.20) | 0.3444 | 1.06 (0.93, 1.20) | 0.3659 | **0.79 (0.61, 1.02)** | **0.0704** |
|  | non-Hispanic Black | 1.00 (0.90, 1.11) | 0.9427 | 1.00 (0.90, 1.11) | 0.9496 | **0.83 (0.68, 0.99)** | **0.0358** |
|  | non-Hispanic other | 0.77 (0.57, 1.03) | 0.0814 | 0.76 (0.57, 1.03) | 0.0745 | 0.77 (0.49, 1.21) | 0.2552 |
|  | Proxy response (yes) | **0.52 (0.47, 0.57)** | **<0.0001** | **0.52 (0.47, 0.57)** | **<0.0001** | **0.52 (0.47, 0.57)** | **<0.0001** |
|  | Year × gender (female) |  |  | 1.01 (0.99, 1.03) | 0.1777 |  |  |
|  | Year × race/ethnicity (Hispanic) |  |  |  |  | **1.03 (1.01, 1.05)** | **0.0113** |
|  | Year × race/ethnicity (non-Hispanic Black) |  |  |  |  | **1.02 (1.00, 1.04)** | **0.017** |
|  | Year × race/ethnicity (non-Hispanic other) |  |  |  |  | 1.00 (0.96, 1.04) | 0.93 |
| Number of unmet IADL support needs | Year | **1.06 (1.05, 1.07)** | **<0.0001** | **1.05 (1.03, 1.08)** | **<0.0001** | **1.07 (1.06, 1.09)** | **<0.0001** |
|  | Age | 1.00 (0.99, 1.01) | 0.9155 | 1.00 (0.99, 1.01) | 0.9135 | 1.00 (0.99, 1.01) | 0.9675 |
|  | Gender (female) | 0.90 (0.78, 1.04) | 0.1558 | 0.81 (0.59, 1.13) | 0.2134 | 0.90 (0.78, 1.04) | 0.1568 |
|  | Race/ethnicity (ref: non-Hispanic White) |  |  |  |  |  |  |
|  | Hispanic | **0.46 (0.37, 0.58)** | **<0.0001** | **0.46 (0.36, 0.58)** | **<0.0001** | 0.82 (0.51, 1.30) | 0.389 |
|  | non-Hispanic Black | **0.71 (0.61, 0.83)** | **<0.0001** | **0.71 (0.61, 0.83)** | **<0.0001** | 1.08 (0.79, 1.49) | 0.6398 |
|  | non-Hispanic other | 0.84 (0.60, 1.20) | 0.3378 | 0.84 (0.59, 1.19) | 0.3295 | 0.91 (0.41, 2.03) | 0.818 |
|  | Proxy response (yes) | **1.32 (1.16, 1.51)** | **<0.0001** | **1.32 (1.16, 1.51)** | **<0.0001** | **1.31 (1.15, 1.49)** | **<0.0001** |
|  | Year × gender (female) |  |  | 1.01 (0.98, 1.04) | 0.5291 |  |  |
|  | Year × race/ethnicity (Hispanic) |  |  |  |  | **0.95 (0.91, 0.99)** | **0.0149** |
|  | Year × race/ethnicity (non-Hispanic Black) |  |  |  |  | **0.96 (0.93, 0.99)** | **0.0061** |
|  | Year × race/ethnicity (non-Hispanic other) |  |  |  |  | 0.99 (0.93, 1.06) | 0.8569 |

Notes. BADL=basic activity of daily living; IADL=instrumental activity of daily living. Trends were measured by adjusted relative ratio (RR) and its 95% confidence interval (CI), which was obtained from the coefficient of the “year” predictor in the Poisson regression, controlling for age, gender, race/ethnicity, and whether need proxy response. RR>1 indicates an increasing trend in the number of unmet BADL or IADL support needs across the study years, and RR<1 the converse.


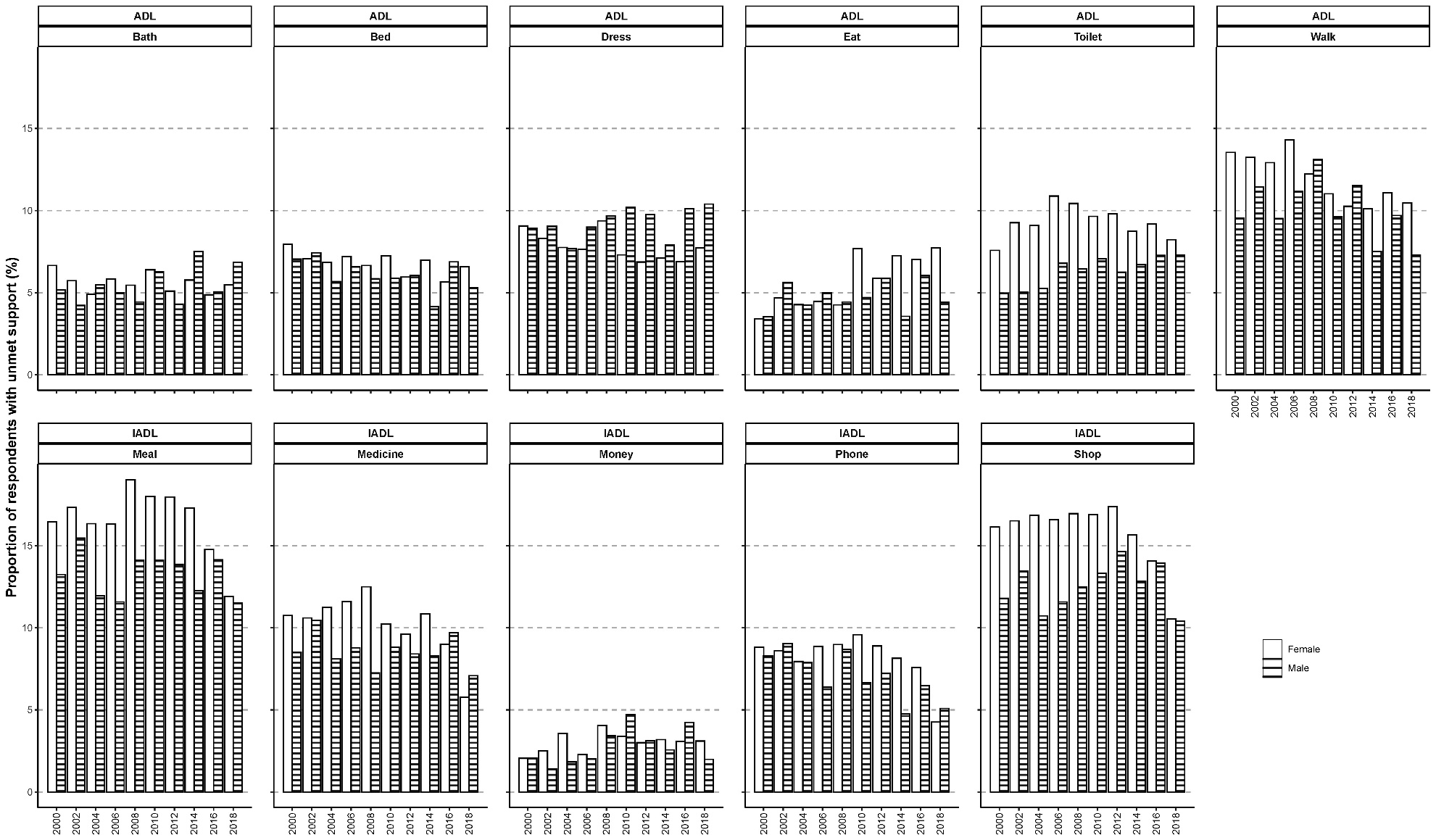


**Supplementary Figure 1. Proportion of respondents with unmet support needs by gender and by BADL and IADL items, among those with CIND or dementia living alone.**

Notes. BADL=basic activity of daily living; IADL=instrumental activity of daily living; CIND=cognitive impairment, no dementia.


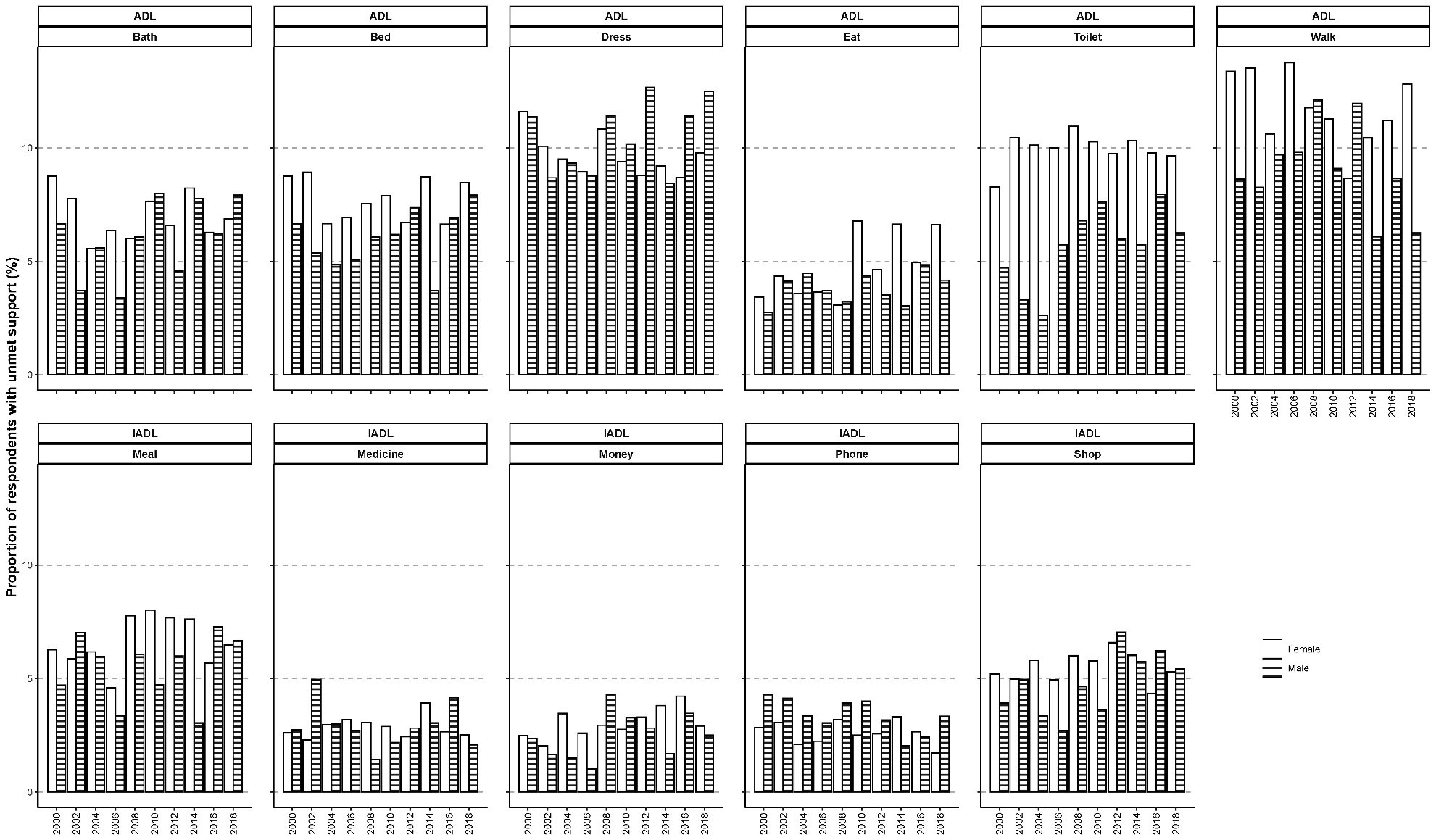


**Supplementary Figure 2. Proportion of respondents with unmet support needs by gender and by BADL and items, among those with CIND living alone.**

Notes. BADL=basic activity of daily living; IADL=instrumental activity of daily living; CIND=cognitive impairment, no dementia.


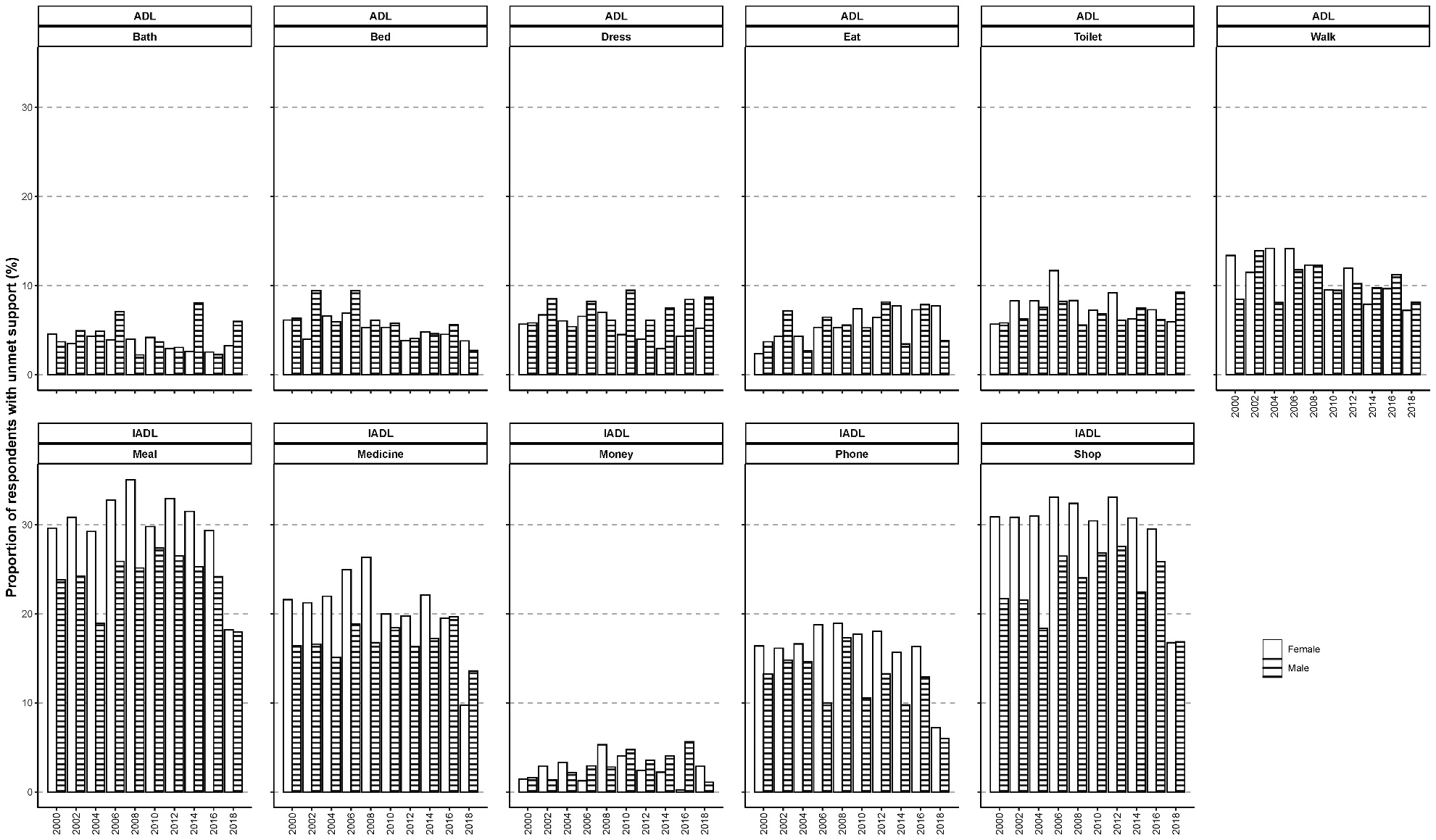
**Supplementary Figure 3. Proportion of respondents with unmet support needs by gender and by items of ADL and IADL, among those with dementia living alone.**

Notes. BADL=basic activity of daily living; IADL=instrumental activity of daily living; CIND=cognitive impairment, no dementia.


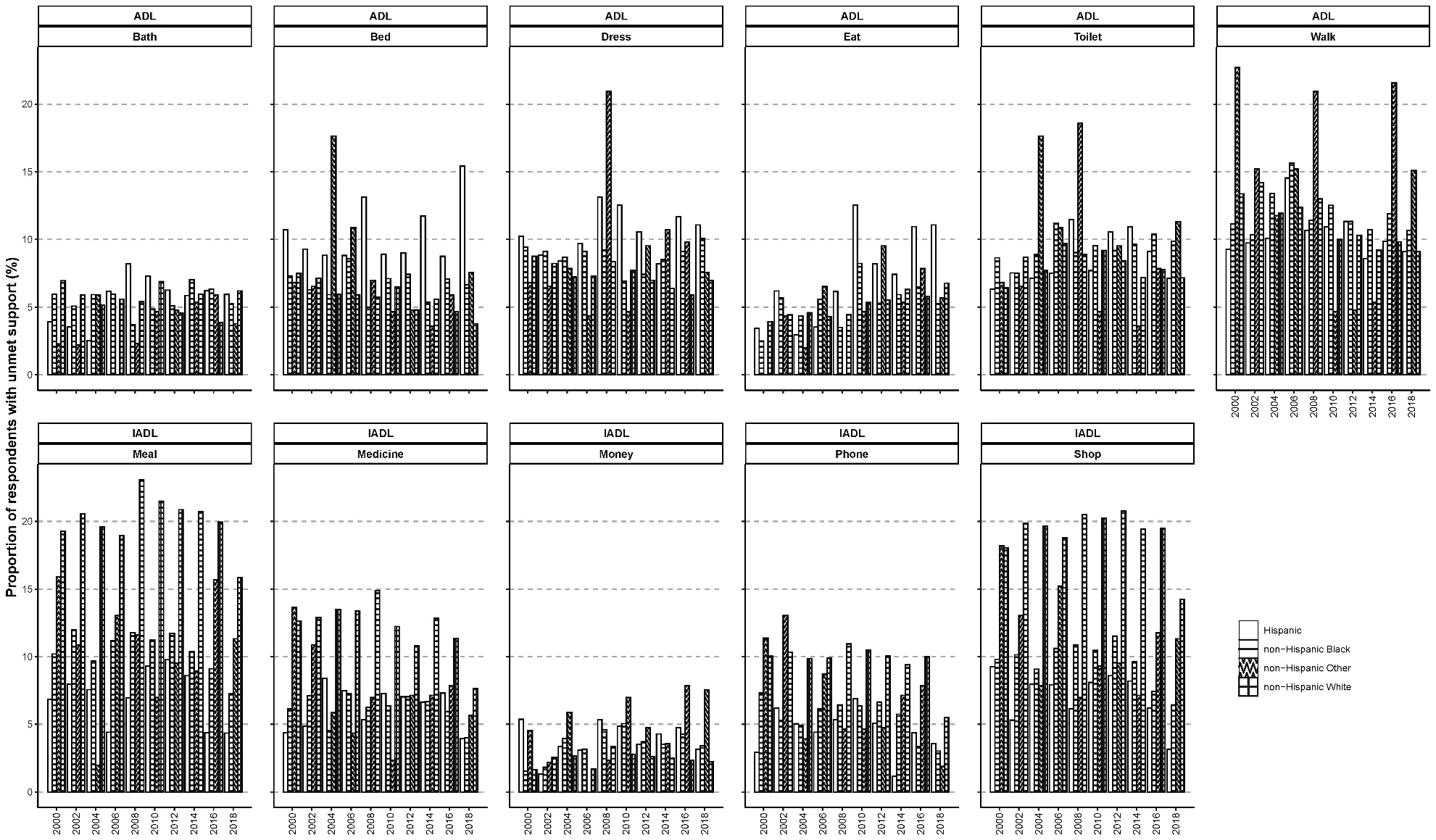


**Supplementary Figure 4. Proportion of respondents with unmet support needs by race/ethnicity and by BADL and IADL items, among those with CIND or dementia living alone.**

Notes. BADL=basic activity of daily living; IADL=instrumental activity of daily living; CIND=cognitive impairment, no dementia.


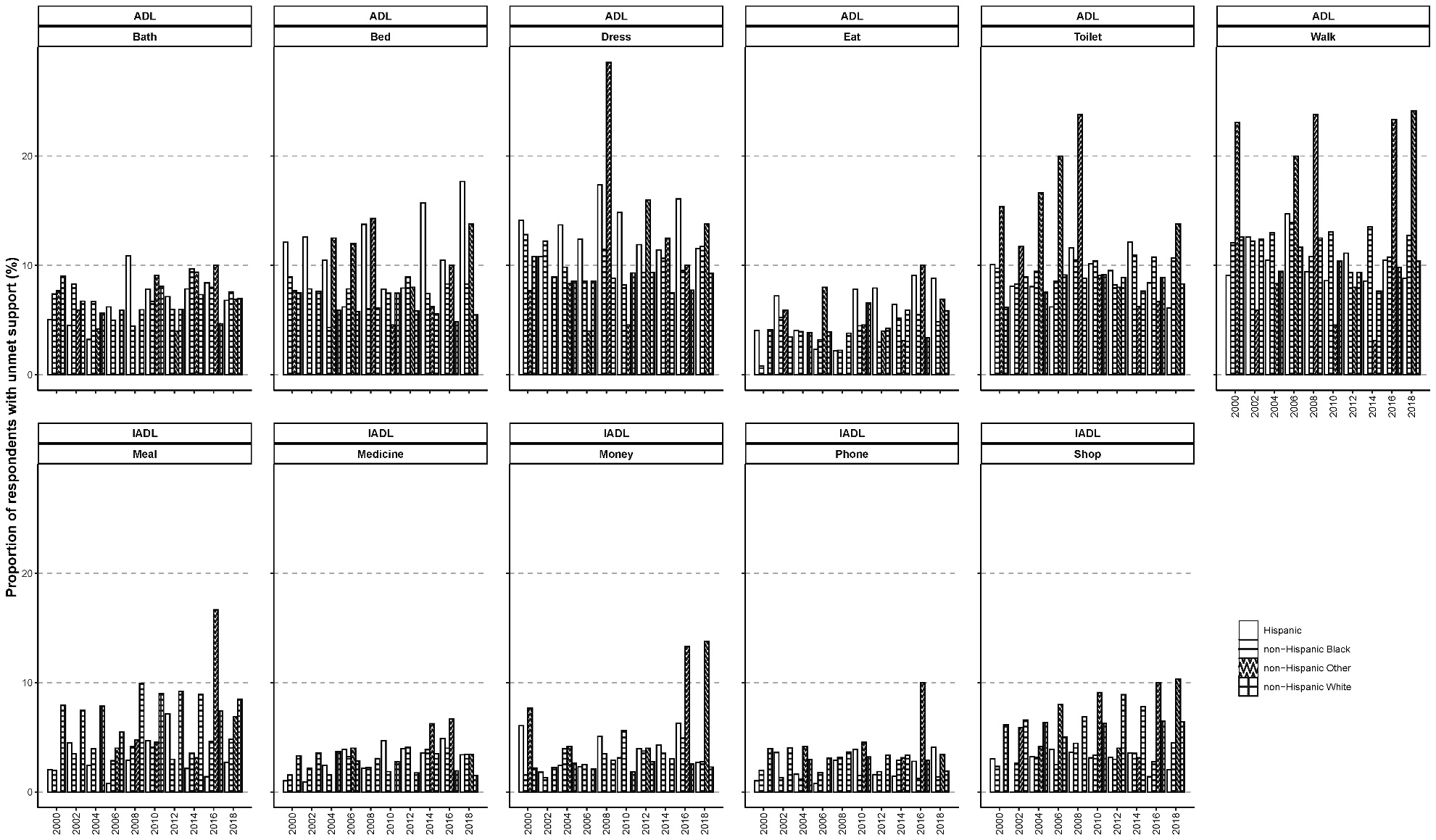
**Supplementary Figure 5. Proportion of respondents with unmet support needs by race/ethnicity and by BADL and IADL items, among those with CIND living alone.**

Notes. BADL=basic activity of daily living; IADL=instrumental activity of daily living; CIND=cognitive impairment, no dementia.


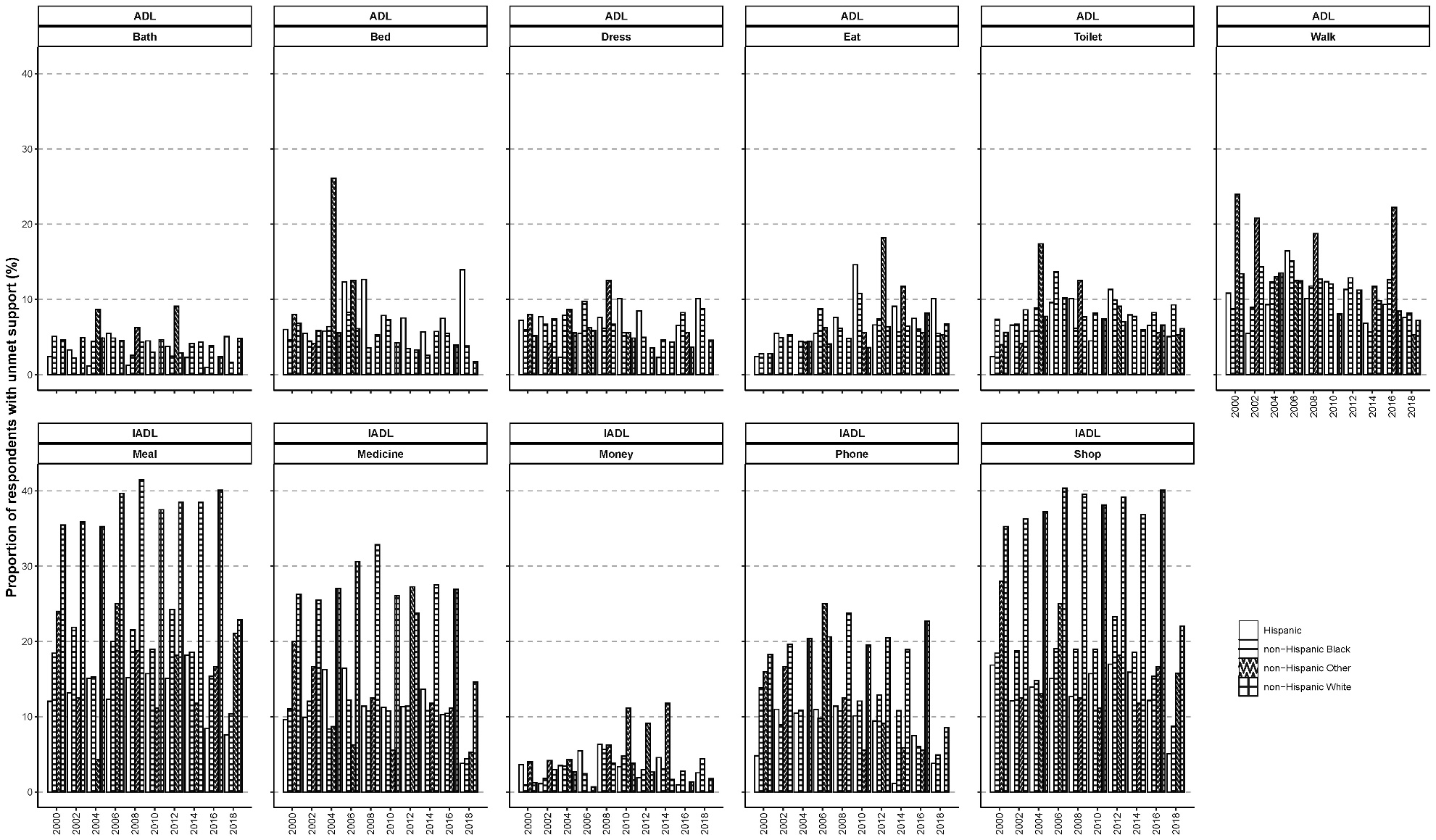
**Supplementary Figure 6. Proportion of respondents with unmet support needs by race/ethnicity and by BADL and IADL items, among those with dementia living alone.**

Notes. BADL=basic activity of daily living; IADL=instrumental activity of daily living; CIND=cognitive impairment, no dementia.


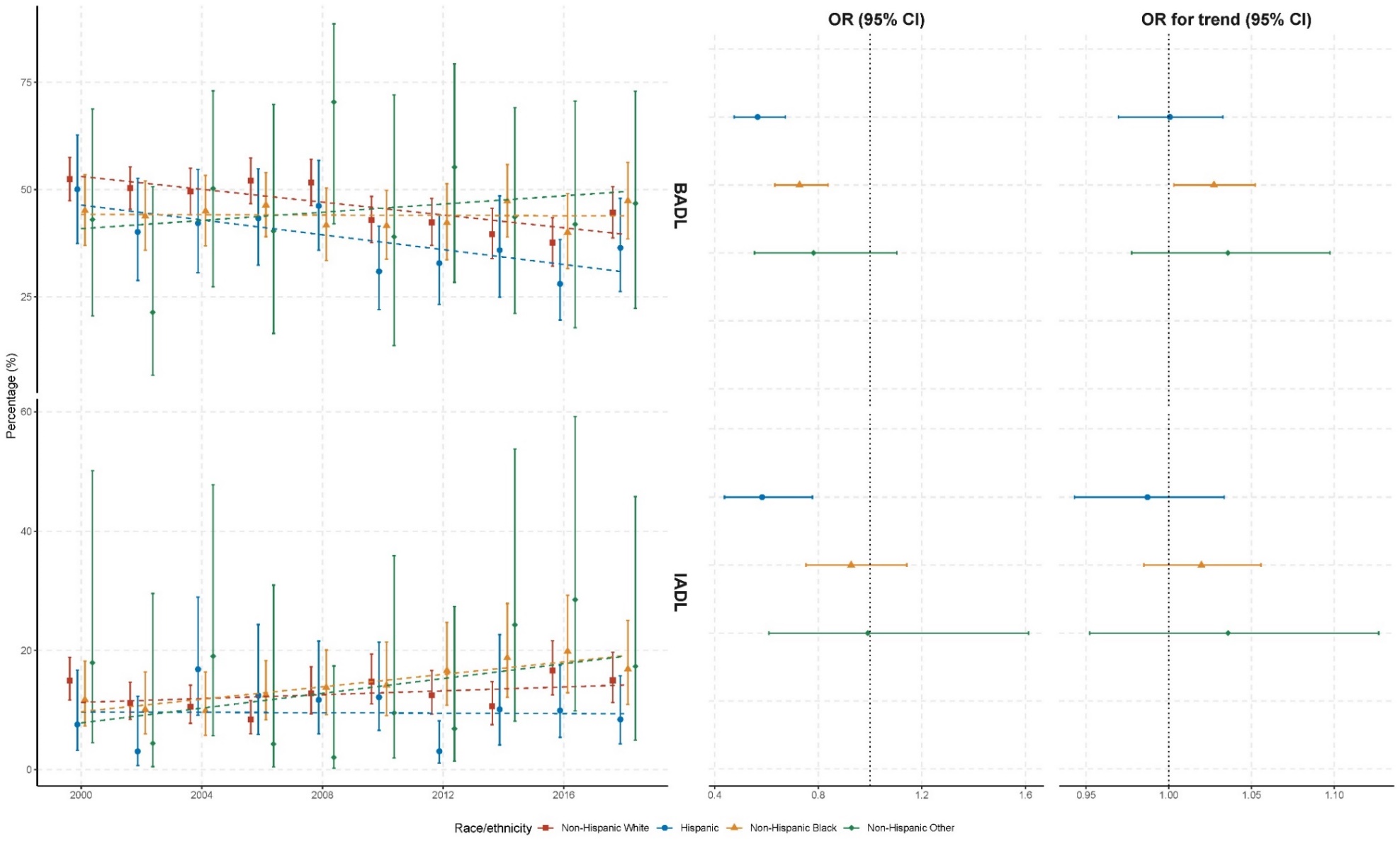


**Supplementary Figure 7. The prevalence of unsupported BADL/IADL disability among cognitively impaired older adults living alone in the US, biennially from 2000 to 2018, by race/ethnicity.**

Notes. The left presents the weighted percentage of having BADL or IADL disability without corresponding support, estimated from raw data, with error bars representing 95% confidence intervals (CI). The dotted lines in the left panel show linear regressions on the weighted percentage of having unsupported BADL or IADL disability. The middle panel shows the race/ethnicity disparities in the probability of having unsupported BADL/IADL disability, measured via the adjusted odds ratio (OR) and its 95% CI, which was obtained from the coefficient for race/ethnicity (relative to the non-Hispanic White reference category) in the logistic regression, controlling for age, gender, whether a proxy response was required, and dementia status. OR for main effects in the middle panel from Model 2 of Table 2. The right panel shows the estimated time trend in the probability of having unsupported BADL/IADL disability. Trends were also measured for the adjusted OR (with 95% CI) from the coefficient for the year × race/ethnicity interaction, controlling for the same covariates. OR for trend interactions from Model 3 of Table 2. A trend OR >1 indicates an increasing trend in the prevalence across the study years, relative to any overall trend, and <1 a decreasing trend. BADL, basic activity of daily living. IADL, instrumental activity of daily living.


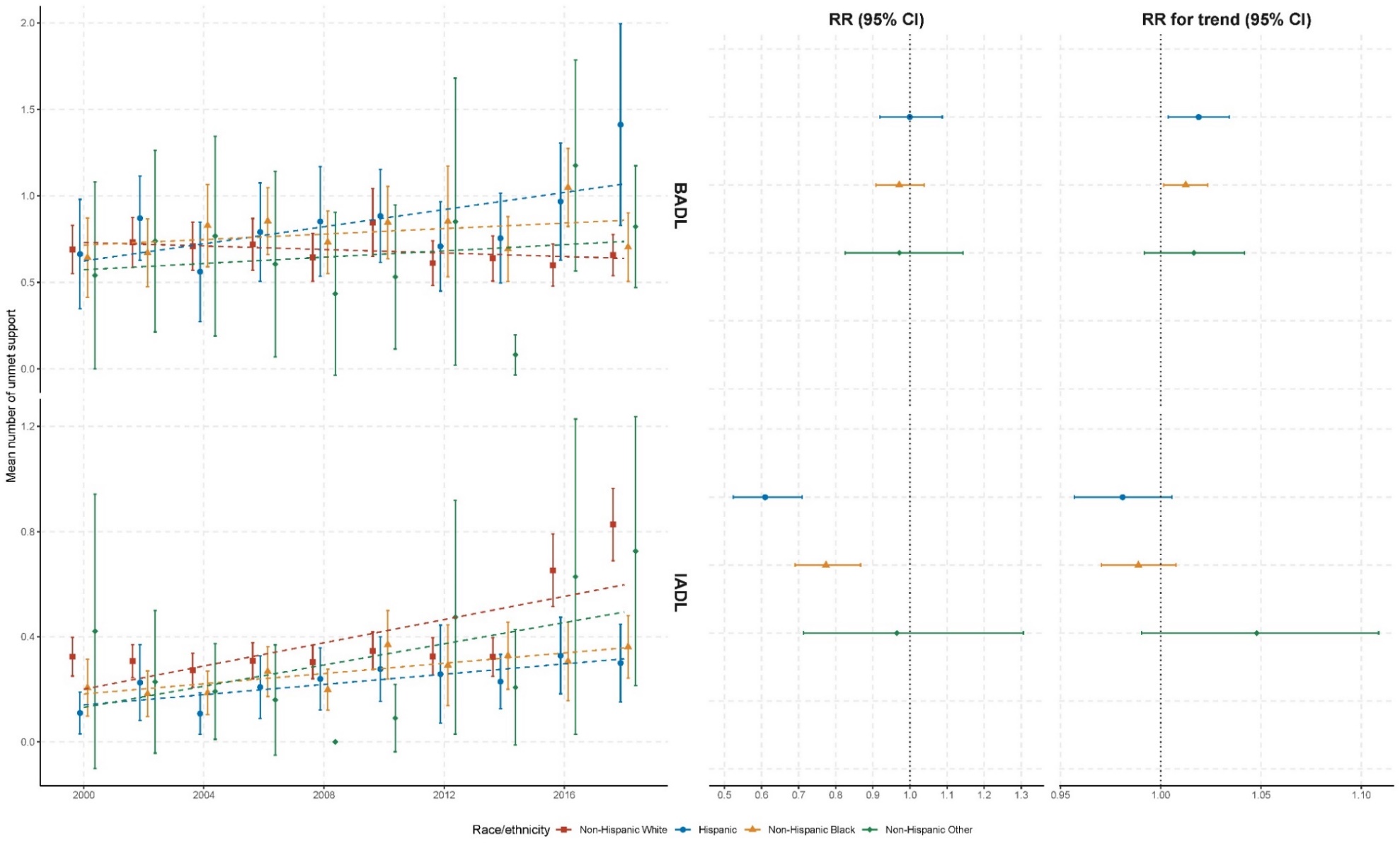


**Supplementary Figure 8.** **The** **number of unmet BADL or IADL support needs among cognitively impaired older adults living alone in the US, biennially from 2000 to 2018, by race/ethnicity.**

Notes. The left panel presents the weighted mean number of unmet BADL or IADL support needs estimated from raw data, with error bars representing 95% confidence intervals (CI). The dotted lines in the left panel show linear regressions on the weighted mean number of unmet BADL or IADL support needs. The middle panel shows the race/ethnicity disparities in the number of unmet BADL or IADL support needs, measured via the adjusted relative ratio (RR) and its 95% CI, which was obtained from the coefficient of the race/ethnicity term (with the non-Hispanic White group as the reference) in the Poisson regression, controlling for age, gender, whether a proxy response was required, and dementia status. RR for main effects in the middle panel from Model 2 of Table 3. The right panel shows the estimated time trend in the number of unmet BADL or IADL support needs. Trends were also measured via the adjusted relative ratio (RR) and its 95% CI, which was obtained from the coefficient of the year × race/ethnicity interaction, controlling for the same covariates. RR for trend interactions from Model 3 of Table 3. A trend RR >1 indicates an increasing trend in the number of unmet BADL or IADL support needs across the study years, relative to any overall trend, and <1 the converse. BADL, basic activity of daily living. IADL, instrumental activity of daily living.
